# Delimitation despite discordance: Evaluating the species limits of a confounding species complex in the face of mitonuclear discordance

**DOI:** 10.1002/ece3.8018

**Published:** 2021-08-11

**Authors:** Thomas J. Firneno, Justin R. O’Neill, Michael W. Itgen, Timothy A. Kihneman, Josiah H. Townsend, Matthew K. Fujita

**Affiliations:** ^1^ Department of Biology University of Texas at Arlington Arlington TX USA; ^2^ Department of Biology, Amphibian and Reptile Diversity Research Center University of Texas at Arlington Arlington TX USA; ^3^ Department of Biology University of Maryland College Park MD USA; ^4^ Department of Biology Colorado State University Fort Collins CO USA; ^5^ Department of Biology Indiana University of Pennsylvania Indiana PA USA; ^6^ Departamento de Ambiente y Desarrollo Centro Zamorano de Biodiversidad Escuela Agrícola Panamericana Zamorano Municipalidad de San Antonio de Oriente Francisco Morazán Honduras

**Keywords:** conservation, macroecological modeling, morphometry, species boundaries, species delimitation

## Abstract

The delimitation of species is an essential pursuit of biology, and proper taxonomies are crucial for the assessment and conservation management of organismal diversity. However, delimiting species can be hindered by a number of factors including highly conserved morphologies (e.g., cryptic species), differences in criteria of species concepts, lineages being in the early stages of the speciation or divergence process, and discordance between gene topologies (e.g., mitonuclear discordance). Here we use a taxonomically confounded species complex of toads in Central America that exhibits extensive mitonuclear discordance to test delimitation hypotheses. Our investigation integrates mitochondrial sequences, nuclear SNPs, morphology, and macroecological data to determine which taxonomy best explains the divergence and evolutionary relationships among these toads. We found that a three species taxonomy following the distributions of the nuclear SNP haplotypes offers the best explanation of the species in this complex based off of the integrated data types. Due to the taxonomic instability of this group, we also discuss conservation concerns in the face of improper taxonomic delimitation. Our study provides an empirical and integrative hypothesis testing framework to assess species delimitation hypotheses in the face of cryptic morphology and mitonuclear discordance and highlights the importance that a stable taxonomy has over conservation‐related actions.

## Graphical abstract

The delimitation of species is an essential pursuit of biology, and proper taxonomies are crucial for the assessment and conservation management of organismal diversity. However, delimiting species can be hindered by a number of factors including highly conserved morphologies (e.g., cryptic species), differences in criteria of species concepts, and discordance between gene topologies (e.g., mitonuclear discordance). Our investigation integrates mitochondrial sequences, nuclear SNPs, morphology, and macroecological data to determine which taxonomy best explains the divergence and evolutionary relationships among a confounded species complex of Central American toads.

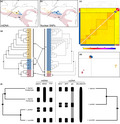

## 1 INTRODUCTION

The delimitation of species is an area of great importance across many fields of biology, especially with respect to understanding the depth and breadth of biodiversity, trait evolution, and/or conservation management. While traditional taxonomic practices used accessible phenetic traits (e.g., morphology and developmental traits) to delimit species, the integration of molecular data has exposed cryptic diversity across many lineages (Bickford et al., 2007). Applications of molecular species delimitation have tended to integrate mitochondrial DNA (mtDNA) and/or a limited number of nuclear DNA (nuDNA) markers, though in recent years large genomic datasets that include hundreds to thousands of loci have become much more commonplace (Fennessy et al., 2016; Hofmann et al., 2019; Thielsch et al., 2017; Victor, 2015). This has provided greater taxonomic resolution for many groups of organisms and has given us a true picture of the evolutionary relationships of species (Funk & Omland, 2003; Spinks et al., 2014). While adding more molecular data to taxonomic studies can be useful, it can also sometimes confound the taxonomic and evolutionary relationships of species even further. This is often demonstrated when you have incongruent evolutionary histories, such as between mitochondrial and nuclear loci (“mitonuclear discordance”; Toews & Brelsford, 2012). Mitonuclear discordance can arise from a number of factors including introgression, incomplete lineage sorting (ILS), and sex‐biased dispersal (Firneno et al., 2020; Funk & Omland, 2003; Ivanov et al., 2018; Phuong et al., 2016; Toews & Brelsford, 2012). While mtDNA markers have proven useful through molecular barcoding for the detection of cryptic species, there have been a number of cases where mitochondrial‐based methodologies conflict with traditional taxonomy and genomic inferences (Toews & Brelsford, 2012). The addition of larger genomic datasets, along with a growing prevalence of discordance between genetic datasets, has caused for the need to incorporate more lines of evidence into species delimitation in an integrative taxonomic framework, as well as the reevaluation of cryptic species and/or confounding species complexes (Padial et al., 2010).

Here, we address a controversial issue of species delimitation in a confounding complex of Central American true toads (Anura: Bufonidae: *Incilius coccifer* complex). Since the taxonomic revision of the species in this complex, which was based on morphology and mtDNA by Mendelson et al. (2005), the nominal species have been the subject of continuous debate and reevaluation as a complex (Firneno & Townsend, 2019; McCranie, 2015; McCranie & Castañeda, 2007; Mendelson et al., 2011). The *I. coccifer* complex is currently comprised of three distinct, yet closely related mitochondrial lineages that exhibit a highly conserved morphology: *I*. *coccifer* in the Pacific lowlands from southern Mexico to northern Costa Rica, *I. ibarrai* in the highlands from western Guatemala to western Honduras, and *I. porteri* in the highlands of central Honduras (Firneno & Townsend, 2019; Mendelson et al., 2005, 2011). The recent addition of genomic SNP data found extensive mitonuclear discordance between the two genetic datasets, which was determined to be the cause of incomplete lineage sorting (ILS) of mitochondrial haplotypes (Firneno et al., 2020). The conflicting genetic evidence has raised a complicated example of species delimitation where a stable and accurate taxonomy is of particular concern, as these organisms have been constantly reassessed as being data deficient, endangered, or least concern by the IUCN Red List of Threatened Species and exist in some of the most disturbed and threatened habitats in Central America (Mendelson et al., 2004; Townsend & Wilson, 2010, 2016; Whitfield et al., 2016).

The mitonuclear discordance found in the *I. coccifer* complex involves differences in tree topology and the distribution of populations/species (Firneno et al., 2020; Firneno & Townsend, 2019; Mendelson et al., 2005, 2011), therefore making it unclear exactly how many species there are in the complex and what their geographic range distribution is. Here we integrate and reevaluate previously collected molecular data (mtDNA—Firneno & Townsend, 2019; Mendelson et al., 2005; genome‐wide SNPs—Firneno et al., 2020), previously and newly collected morphology (Mendelson et al., 2005), and macroecological modeling in a robust statistical and comparative integrative taxonomic framework. We use three hypotheses to test and reevaluate the species limits of the *I. coccifer* complex in the face of mitonuclear discordance: (a) there are three species in the complex that follow the distributions proposed in Mendelson et al. (2005) according to mtDNA and morphological data (currently recognized taxonomy); (b) there are three species in the complex that follow the distributions proposed in Firneno et al. (2020) according to SNP data and biogeographic barriers of the region; or (c) there are some other number of species in the complex that will be revealed by integrating molecular, morphological, and macroecological data that have different range distributions than the currently proposed ones. We test these hypotheses and define species in this paper using the general lineage concept (GLC), due to its pluralistic approach and the ability to use numerous criteria to define a species under its definition (Mayden, 1997; Queiroz, 1998, 1999; Sangster, 2014). Our study highlights the importance of using multiple genetic markers and multiple lines of evidence in an integrative hypothesis testing framework to resolve complex species delimitation scenarios where different lines of evidence may conflict, as well as the importance of a stable taxonomy to guide proper conservation assessment measures.

## 2 MATERIALS AND METHODS

### 2.1 Molecular data generation and processing

We reprocessed and reanalyzed the ddRADseq data of Firneno et al. (2020) (available on GenBank as PRJNA626342: SAMN14615759–SAMN14615822). The workflow for data processing, filtering, and formatting was automated using an updated pipeline available from https://github.com/dportik/Stacks_pipeline (Portik et al., 2017). In brief, the raw Illumina reads were demultiplexed using stacks v2.53 (Catchen et al., 2013), the restriction site overhangs were removed using the fastx_trimmer module of the fastx‐toolkit (www.hannonlab.cshl.edu/fastx_toolkit), and the sequencing quality was examined on a per sample basis using fastqc v0.10.1 (www.bioinformatics.babraham.ac.uk/projects/fastqc). Loci were created, catalogued, and identified using ustacks, cstacks, gstacks, and sstacks, respectively. populations was then used to generate alleles for loci present in 80% of all individuals, which resulted in 3,225 loci. Custom filtering removed “blank” loci (*n* = 87), invariant loci (*n* = 1,120), nonbiallelic loci (*n* = 0), and loci containing at least one individual with more than two alleles (*n* = 0). For loci containing multiple SNP sites, we randomly chose a single SNP to be used for subsequent analyses. Samples missing data for more than 50% of loci were removed. After completing the above filtering steps, our final SNP dataset consisted of 64 samples and 2,018 loci.

**FIGURE 1:**
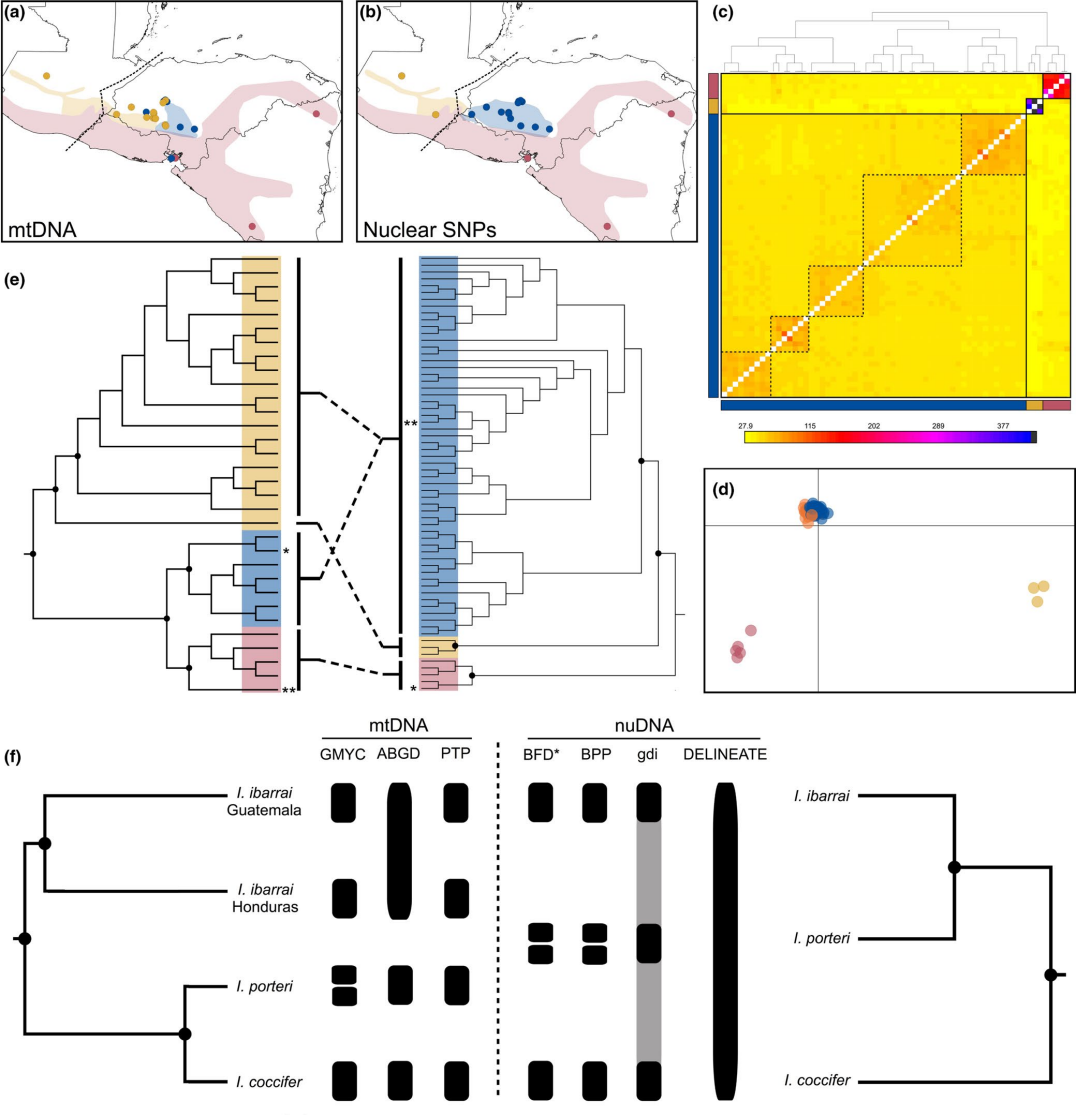
Distribution of haplotypes in the *I. coccifer* complex (*I. coccifer* in red, *I. ibarrai* in yellow, and *I. porteri* in blue) and sampling for our (a) mitochondrial and (b) SNP datasets. The Chortís/Guatemala Highland boundary is indicated by the dashed line. (c) Coancestry matrix from fineradstructure based on our SNP dataset (coefficients of coancestry are color‐coded from low (yellow) to high (black), and the dendrogram depicts a clustering of individual sampled based on the pairwise matrix of coancestry coefficients) indicating main populations (bolded squares) with substructuring identified within the sampling east of the Chortís/Guatemala Highlands boundary (dashed squares). (d) Discriminant analysis of principal components of SNP dataset. (e) Maximum likelihood cladograms of mtDNA (left) and SNP (right) datasets exhibiting extent of mitonuclear discordance. Nodes with high support (bootstrap ≥90) are indicated by black dots, and asterisks (*) indicate samples with haplotypes that do not follow the exact pattern of mitonuclear discordance shown by dashed lines. (f) Summary of species delimitation analyses, with collapsed cladogram inferred from BEAST analysis of mtDNA (left) and SNAPP species tree of SNP dataset (right). Nodes with high support (posterior probability ≥0.9) are indicated by black dots. Blocks represent species units that have been estimated for each analysis. Gray areas connecting blocks indicate that the support for the split between those taxa is low to moderate (*gdi* between 0.1 and 0.7)

We also created a complimentary mtDNA dataset of the cytochrome oxidase I (COI) gene using available sequences on GenBank (Firneno & Townsend, 2019; Firneno et al., 2020). Individuals in this dataset either had complementary RADseq data associated with them or were from roughly the same sampling sites (Table S1; Figure 1a,b). We generated a multiple alignment of 610 bp for 59 samples (including two outgroups; Table S1) in mega7 (Kumar et al., 2016) with the muscle algorithm (Edgar, 2004) using the default parameters.

### 2.2 Population structure

We determined the number of discrete populations present across the sampled range of the *I. coccifer* complex with our RADseq dataset using a combination of Bayesian and likelihood clustering analyses and multivariate methods. We used structure v2.3.4 (Falush et al., 2003; Pritchard et al., 2000) to examine the number of population clusters and potential admixture between populations in our dataset using MCMC. Hierarchical analyses were performed for 10 runs per K, up to a maximum of six populations, and used the admixture model with a burn‐in of 10,000 steps followed by 100,000 steps. We summarized our results using structureharvester (Earl & vonHoldt, 2012) and evaluated the number of populations based on inspection of likelihood plots and following Evanno et al. (2005). To complement our structure analysis, we used a maximum likelihood approach with admixture (Jombart, 2008). We performed ten replicate analyses to evaluate up to seven populations. To assess the best *K* value, we performed 10‐fold cross‐validation and determined the *K* value with the lowest cross‐validation error. We also evaluated the number of discrete populations using a discriminant analysis of principal components (DAPC) with adegenet v2.0.0 (Jombart, 2008; Jombart & Ahmed, 2011). A maximum of 10 clusters were investigated using the k‐means algorithm. The preferred number of clusters was evaluated using BIC scores. We explored a range of three to five clusters to describe using DAPC. To minimize overfitting, an initial DAPC was used to find the a‐score for each set of clusters and this value was used to select the number of principal components to retain in a subsequent reanalysis (Jombart, 2008; Jombart & Ahmed, 2011). Group membership probabilities were then examined for each cluster. To independently assess the validity of population differentiation and assignment, we used the fineradstructure software package (Malinsky et al., 2018) to construct a coancestry matrix from our RADseq data. We used a 100,000 burn‐in followed by 100,000 MCMC steps sampling every 1,000 steps, and the tree was constructed with 10,000 hill‐climbing iterations. The results were visualized using the FINERADSTRUCTUREPLOT.R and FINESTRUCTURELIBRARY.R scripts (included in the fineradstructure package file).

### 2.3 Phylogenetic analyses

We estimated the phylogenetic relationships independently for our mtDNA and SNP datasets. We used a maximum likelihood approach carried out in RAxML v8.0 (Stamatakis, 2014) on both genetic datasets with 1,000 bootstrap replicates under a GTR substitution model. We used a Bayesian approach for our mtDNA dataset by employing beast v2.5.1 (Bouckaert et al., 2014) using an HKY model of nucleotide substitution, relaxed clock model, coalescent constant population, and a random starting tree with a Markov Chain Monte Carlo (MCMC) run for 2 × 10^7^ generations, sampling every 1,000 generations producing a total of 10,000 trees. We assessed the run using Tracer v1.6 (Rambaut & Drummond, 2009) to examine convergence. A burn‐in of 10% was discarded, and a maximum clade credibility tree with median heights was created from the remaining 9,000 trees. We then estimated the species tree for our SNP dataset using snapp v1.3.0 (Bryant et al., 2012) implemented in beast2. To reduce run times, we subsampled each population to include 3–6 representatives, for a total of 20 individuals. We estimated the mutation rates (*u* and *v*) from the data (1.06 and 0.94, respectively) within beauti. We assigned a gamma distribution to our birth rate (λ) of the Yule prior, with and alpha of 2.0 and a beta of 2.0. Our snapp prior was assigned an alpha of 11.75, a beta of 109.73, and a lambda of 0.01. We performed two independent runs with a chain length of 1,000,000 generations, sampling every 1,000 generations. Runs were assessed using tracer v1.6 (Rambaut & Drummond, 2009) to examine convergence, and tree topologies and node heights were visualized using densitree (Bouckaert, 2010).

### 2.4 Species delimitation

To estimate the best‐fit number of species units based on our two genetic datasets, we used a suite of different species delimitation analyses. We applied three single locus delimitation analyses to our mtDNA dataset including (a) the General Mixed Yule Coalescent (GMYC) model, which establishes thresholds between the branching patterns of ultrametric gene trees from inter‐ and intraspecific branches in order to define speciation events (Fujisawa & Barraclough, 2013); (b) the Poisson Tree Process (PTP; Kapli et al., 2016), which, like GMYC, aims to identify the transition between inter‐ and intraspecific processes, but specifically requires that the phylogenetic gene trees branch lengths are proportional to the number of substitutions, rather than to time as for GMYC; (c) and the Automated Barcode Gap Discovery (ABGD) method (Puillandre et al., 2012), which partitions sequences into groups based on comparisons of pairwise distances and compared to tree‐based delimitation methods (GMYC and PTP) that are suspected to over split, ABGD offers a more conservative approach to estimate the number of species given comprehensive sampling (Puillandre et al., 2012). For the GMYC analysis, we applied a single model using the R package *splits* to our ultrametric tree generated in BEAST. Next, we implemented the bPTP analysis using our maximum likelihood tree on the bPTP server (https://species.h‐its.org; Zhang et al., 2013). For this analysis, we ran 100,000 MCMC generations, with a thinning of 100 and burn‐in of 0.1. Finally, we applied the AGBD analysis to our data using the web interface (https://bioinfo.mnhn.fr/abi/public/abgd). For our analysis of each locus dataset, we used the default maximum intraspecific distance values (*P*_max_ = 0.1), a relative barcode gap width of *X* = 1.5, and a distance correction of Jukes‐Cantor (JC69). Default settings were used for all of the remaining parameters.

We then applied four species delimitation methods to our SNP dataset including (a) Bayes Factor Delimitation (BFD*; Leaché et al., 2014) and (b) Bayesian Phylogenetics and Phylogeography (BPP) v4.2 (Flouri et al., 2018), both of which implement the multispecies coalescent (MSC) model; (c) heuristic species delimitation using the genealogical divergence index (*gdi*) to determine whether putative species boundaries correspond to species‐level divergences between populations; and (d) delineate, which implements the protracted speciation model (PSM) to account for the assertion that the MSC delimits populations rather than species (Sukumaran et al., 2021). We conducted Bayes Factor Delimitation implemented in snapp v1.3.0 (Bryant et al., 2012) in beast2 v2.5.4 (Bouckaert et al., 2014) with our SNP dataset following Leaché et al. (2014; e.g., implementing BFD*). We performed our analyses testing four models, which are presented in Table S2. We compared and ranked models to select the best‐supported species hypothesis. We calculated the Bayes Factor (BF) by subtracting the value of the log MLE for the model representing the current taxonomic classification from each alternative model and multiplying the difference by two (BF = 2(model 1 − model 2)). We ranked all of the models and selected the model with the highest BF (Table 1).

**TABLE 1:** Classification rates of the linear discriminant function analysis

Taxonomic ID	Delimitation hypothesis—two species				
	Nuclear SNP assignment				
	Lowland	Highland	—	—	Total
Lowland	**130 (94.9%)**	7	—	—	137
Highland	22	**225 (91.1%)**	—	—	247
					384

	mtDNA assignment				
	Lowland	Highland	—	—	Total
Lowland	**130 (94.9%)**	7	—	—	137
Highland	22	**225 (91.1%)**	—	—	247
					384

	Delimitation hypothesis—three species				
	Nuclear SNP assignment				
	*I. coccifer*	*I. ibarrai*	*I. porteri*	—	Total
*I. coccifer*	**130 (94.9%)**	4	4	—	138
*I. ibarrai*	0	**92 (89.3%)**	11	—	103
*I. porteri*	22	27	**94 (65.7%)**	—	143
					384

	mtDNA assignment				
	*I. coccifer*	*I. ibarrai*	*I. porteri*	—	Total
*I. coccifer*	**130 (94.9%)**	3	4	—	137
*I. ibarrai*	7	**122 (81.9%)**	20	—	149
*I. porteri*	15	25	**58 (58.2%)**	—	98
					384

	Delimitation hypothesis—four species				
	Nuclear SNP assignment				
	*I. coccifer*	*I. ibarrai*	*I. porteri* C/E	*I. porteri* W	Total
*I. coccifer*	**131 (94.9%)**	1	4	2	138
*I. ibarrai*	0	**85 (92.4%)**	4	3	7
*I. porteri* C/E	19	18	**83 (61.5%)**	15	52
*I. porteri* W	2	0	7	**10 (52.6%)**	9
					384

	mtDNA assignment				
	*I. coccifer*	*I. ibarrai* GT	*I. ibarrai* HN	*I. porteri*	Total
*I. coccifer*	**131 (94.9%)**	2	1	4	138
*I. ibarrai* GT	0	**85 (91.4%)**	5	3	93
*I. ibarrai* HN	10	3	**31 (60.8%)**	7	51
*I. porteri*	11	14	9	**68 (66.7%)**	102
					384

We used the same parameters as our snapp analysis (see above). We performed 48 path sampling steps, with 100,000 MCMC generation and a preburn‐in of 1,000 generations. The BF was then calculated for each alternative model, the models were ranked, and a best model was chosen (Table S2).

Next, we used Bayes species delimitation as implemented in BPP v4.2 (Flouri et al., 2018, 2020). We implemented the A10 (species delimitation using a fixed guide tree) analysis under the MSC accommodating introgression (MSCi) model using relationships derived from phylogenetic analyses as a guide tree. The mapping of individuals to their identifiers was done in the *imap* file supplied to BPP. Diffuse inverse‐gamma priors were designated as follows: θ IG(3, 0.02) with mean 0.02/(3 − 1) = 0.01, and τ IG(3, 0.01) with mean 0.01/(3 − 1) = 0.005. Each analysis was run three times for 100,000 generations with the first 10,000 discarded as burn‐in. Convergence was assessed by ensuring the stability of parameter estimates.

Following this, we used the *gdi* to determine whether putative species boundaries correspond to species‐level divergences between populations of the *I. coccifer* complex [25–26]. We calculated *gdi* values in BPP v4.2 (Flouri et al., 2018, 2020) under the MSCi model. The MSCi approach provides a measure of uncertainty in population estimates, and *gdi* can be calculated using the posterior distributions for θ and τ (Leaché et al., 2019). The posterior probability distributions for θ and τ were estimated in the A00 analysis (Jackson et al., 2017) using a fixed species tree containing our three lineages (*I. coccifer*, *I. ibarrai*, and *I. porteri*, based on SNP haplogroups). We used the same diffuse inverse‐gamma priors for θ and τ that we used in our BPP species delimitation analysis (see above). To assess convergence, we compared the posterior distributions from three independent runs (same parameters as BPP above). We then calculated *gdi* for each species by combining all samples from the posterior distributions using the equation: *gdi* = 1 − e^−2τ/θ^ (Chan & Grismer, 2019; Leaché et al., 2018). Lineages were considered distinct species when *gdi* > 0.7, the same species when *gdi* < 0.2, and/or ambiguous species status if 0.2 ≥ *gdi* ≤ 0.7 (Jackson et al., 2017; Pinho & Hey, 2010).

Finally, we used delineate (Sukumaran et al., 2021) to determine the number of species that are within the *I. coccifer* complex under the PSM. We used the species tree from our SNAPP analysis as a guide tree, and within our control file, we constrained the *I. coccifer* lineage (based on previous descriptions of morphological and ecological distinctiveness) but left the *I. ibarrai* and *I. porteri* (highland) lineages as unconstrained to determine if they are separate species from the *I. coccifer* or each other.

### 2.5 Morphological data acquisition and processing

To determine which genetic species assignment (mtDNA or SNP) and species delimitation model yields more morphological differentiation between species, with special attention paid to discordant highland populations, we collected new measurements from 37 specimens of highland population individuals and combined these data with the measurements of Mendelson et al. (2005) for a total dataset of 386 individuals. The morphological traits measured were snout–vent length (SVL), tibial length (TL), hindfoot length (FL), head length (HL), head width (HW), diameter of tympanum (DT), supratympanic crest length (SC), paratoid length (PL), and parotoid width (PW). All measurements were taken with digital calipers and recorded to the nearest 0.1 mm. A principal component analysis was done using the scaled morphological data. We visualized the clustering of two, three, and four species, as well as the discordant assignments of *I. ibarrai* and *I. porteri* using the mtDNA and SNP assignments based on our molecular delimitation results. PCAs were done in program R using the *princomp* function (R Core Team, 2019) and were visualized using the R package *ggplot2* (Wickham, 2016).

We used linear discriminant function analyses (DFA) to determine the extent of morphological distinction among the species. Six analyses were conducted using the classifications of the nuclear and mitochondrial phylogenies due to the mitonuclear discordance seen in highland populations (*I. ibarrai* and *I. porteri*). We log‐transformed the dataset and removed highly correlated traits (≥0.95). Our final morphological dataset for the DFAs included FL, HL, HW, DT, SC, PL, and PW. The DFAs were done in R using the *lda* function in package *MASS* (Venables & Ripley, 2002).

We further investigated the morphological variation among discordant highland populations using redundancy analyses (RDA) and variation partitioning (VP). All of the nontransformed morphological traits were included in these analyses. Taxonomic assignments based on the mtDNA and nuDNA phylogenies were scored for each individual as dummy variables and were treated as the explanatory variables. The VP analysis was used to calculate the explanatory contributions for each phylogeny. A permutation test with 1,000 permutations was then used to determine the significance of the conditional effects for the mtDNA and nuDNA species assignments. The RDA and VP analyses were done in program R v.3.2.2 using the *rda* and *varpart* functions in the package *vegan* (Oksanen et al., 2017; R Core Team, 2019).

### 2.6 Species distribution modeling

To determine the extent of macroecological differentiation between genetically discordant populations, we created species distribution models (SDMs) for all three species based on their distributions according to their respective genetic datasets. We compiled all available museum locality information from vertnet (Constable et al., 2010) and our own sampling for toads of the *I. coccifer* complex. We created two datasets corresponding to our genetic hypotheses (mtDNA or SNP) and assigned species into their respective genetic datasets either based on genetic confirmation or being within the distribution of a genetic haplotype. This resulted in 410 records that were used to create SDMs (Table S3). To reduce spatial autocorrelation of our occurrence dataset, we thinned points by a distance of 1 km (Aiello‐Lammens et al., 2015; Veloz, 2009). This reduced our datasets to the following for each species: mtDNA—*I. coccifer* = 43, *I. ibarrai* = 41, *I*. *porteri* = 30; SNP—*I. coccifer* = 48, *I. ibarrai* = 18, *I. porteri* = 50.

To construct our environmental layer dataset, we extracted nineteen bioclimatic variables from the WorldClim 2.1 database (http://worldclim.org; Fick & Hijmans, 2017) and eighteen environmental variables from the envirem database (https://envirem.github.io/; Title & Bemmels, 2018) for present‐day conditions (~1970–2020). We also included layers of spatial homogeneity of global habitat (http://earthenv.org/texture.html) and global percent of tree cover (https://github.com/globalmaps/gm_ve_v1), and we computed aspect and slope layers within arcgis from the Global 30 Arc‐second digital elevation layer (GTOPO30; https://www.usgs.gov/centers/eros/science/usgs‐eros‐archive‐digital‐elevation‐global‐30‐arc‐second‐elevation‐gtopo30). Spatial resolution for all environmental layers was 30 arc‐seconds (~1 km). To eliminate predictor collinearity among our bioclimatic and envirem variables, we calculated Pearson's correlation coefficients for all pairs of variables in their respective datasets using ENMtools (Warren et al., 2019), excluding the variable from a correlated pair (|*r*| > 0.75) that we considered to be less biologically important based on known preferences of *Incilius* toads (Figure S3). The resulting dataset contained seven bioclimatic variables (BIO1 = annual mean temperature; BIO2 = mean diurnal range; BIO3 = isothermality; BIO12 = annual precipitation; BIO15 = precipitation seasonality; BIO18 = precipitation of warmest quarter; and BIO19 = precipitation of coldest quarter) and six envirem variables (annual potential evapotranspiration (PET), thermicity index, climatic moisture index, aridity index, terrain roughness index, and topographic wetness), bringing our final dataset to a total of seventeen variables. We constrained our environmental variable layers to the range spanned by all of our occurrence records.

To build SDMs, we used maxent (Phillips & Dudík, 2008) implemented in the ecological niche modeling application wallace (Kass et al., 2018). Because model settings can hold strong influence on model output, determining optimal model complexity is important (Radosavljevic & Anderson, 2014; Warren & Seifert, 2011). We ran 76 candidate models using a combination of (a) one of four selected maxent feature classes (Linear; Linear and Quadratic; Hinge; Linear, Quadratic, and Hinge), and (b) a range of 19 regularization multipliers (1–10 in 0.5 increments), allowing our candidate models to range from simple to complex. To test the accuracy of the models, we used wallace’s spatial partition option to split the occurrence data into four spatially independent k‐folds for cross‐validation, whereas, for datasets with under 20 records, we used the jackknife approach because of its accuracy and utility with small datasets (Kass et al., 2018; Muscarella et al., 2014; Shcheglovitova & Anderson, 2013). To choose the top model for each species, we approached it in a hierarchical fashion by (a) looking for the candidate within four ΔAICc that had the lowest testing omission rate at the 10th percentile training presence threshold; (b) if there was a tie at the 10th percentile training presence, we used the difference between the training and testing AUC, picking the model with lowest value; (c) if there was a tie at this value, we used the testing AUC score; and (d) if there was a tie at this value, we used the lowest ΔAICc (Burnham & Anderson, 2002; Kass et al., 2018; Muscarella et al., 2014). Models were reclassified into binary files of suitable and nonsuitable habitat based on the 10th percentile training presence threshold, essentially removing the lowest 10% of the prediction values (Kass et al., 2018).

We inferred the amount of niche overlap between species for the mtDNA distribution and the SNP distribution SDMs using by calculating Schoener's *D* and *I* by pairwise comparison using the *nicheOverlap* functions in the R package *dismo* (Hijmans et al., 2011). These metrics give an output value from 0 to 1, where a value of 0 indicates no overlap between niches/low niche similarity and a value of 1 indicates that the niches completely overlap/are identical.

## 3 RESULTS

### 3.1 Genetic and species delimitation analyses

In agreement with previous studies (Firneno et al., 2020), we see extensive mitonuclear discordance among our two genetic datasets of individuals within the *Incilius coccifer* complex. We estimated four populations from our SNP dataset, with a single lowland population, a highland population that exist west of the Chortís/Guatemala Highlands boundary, and two populations with admixture east of the Chortís/Guatemala Highlands boundary (Figures 1d and S1). Our fineradstructure analysis identified three main populations (as defined above) with some substructuring among individuals in the population east of the Chortís/Guatemala Highlands boundary, which may be due to nuclear introgression or isolation‐by‐distance due to the mosaic nature of the organisms’ highland distributions (Figure 1c).

The single locus delimitation methods (GMYC, PTP, and ABGD) of our mtDNA dataset all yielded varied results in terms of the number of species units they estimated. GMYC estimated five species units, bPTP estimated four species units, and ABGD estimated three species units (Figure 1f). Our MSC‐based methods, BFD* and BPP, both estimated four species units. Our heuristic method (*gdi*) had ambiguous support (0.2 ≥ *gdi* ≤ 0.7) for *I. coccifer* and *I. ibarrai* as distinct species and low support (0.2 ≥ *gdi*) for *I. porteri* as a distinct species (Figure S2), indicating that there may be fewer than three species in the complex. Finally, our PSM‐based method (delineate) estimated one species unit (Figure 1f, Table S2).

Though ML and Bayesian analyses of both datasets yielded different tree topologies, both showed high support (bs ≥ 90/pp ≥ 0.9) for three distinct lineages (Figure 1e,f). Like previous studies, we see a distinct lowland clade (*I. coccifer*) that is the sister group to the two highland lineages according to our SNP dataset, but is nested within the highland clades according to our mtDNA dataset. We also see two highland lineages according to both datasets (*I. ibarrai* and *I. porteri*); however, the distributions of the respective haplotypes are discordant. Our mtDNA dataset identifies one highland clade solely within central Honduras (*I. porteri*) and another clade that spans from central Honduras to western Guatemala (*I*. *ibarrai*), whereas our SNP dataset identifies one highland clade east of the Chortís/Guatemalan Highland boundary (*I. porteri*) and the other west of the Chortís/Guatemalan Highland boundary (*I*. *ibarrai*) (Figure 1a‐b,e). The SNP highland clade that is distributed west of the Chortís/Guatemalan Highland boundary corresponds to a single, divergent sample of *I. ibarrai* that was collected from Guatemala and has previously been identified as possibly being a separate entity from those collected in Honduras (Firneno & Townsend, 2019; Mendelson et al., 2011).

### 3.2 Morphological analyses

**FIGURE 2:**
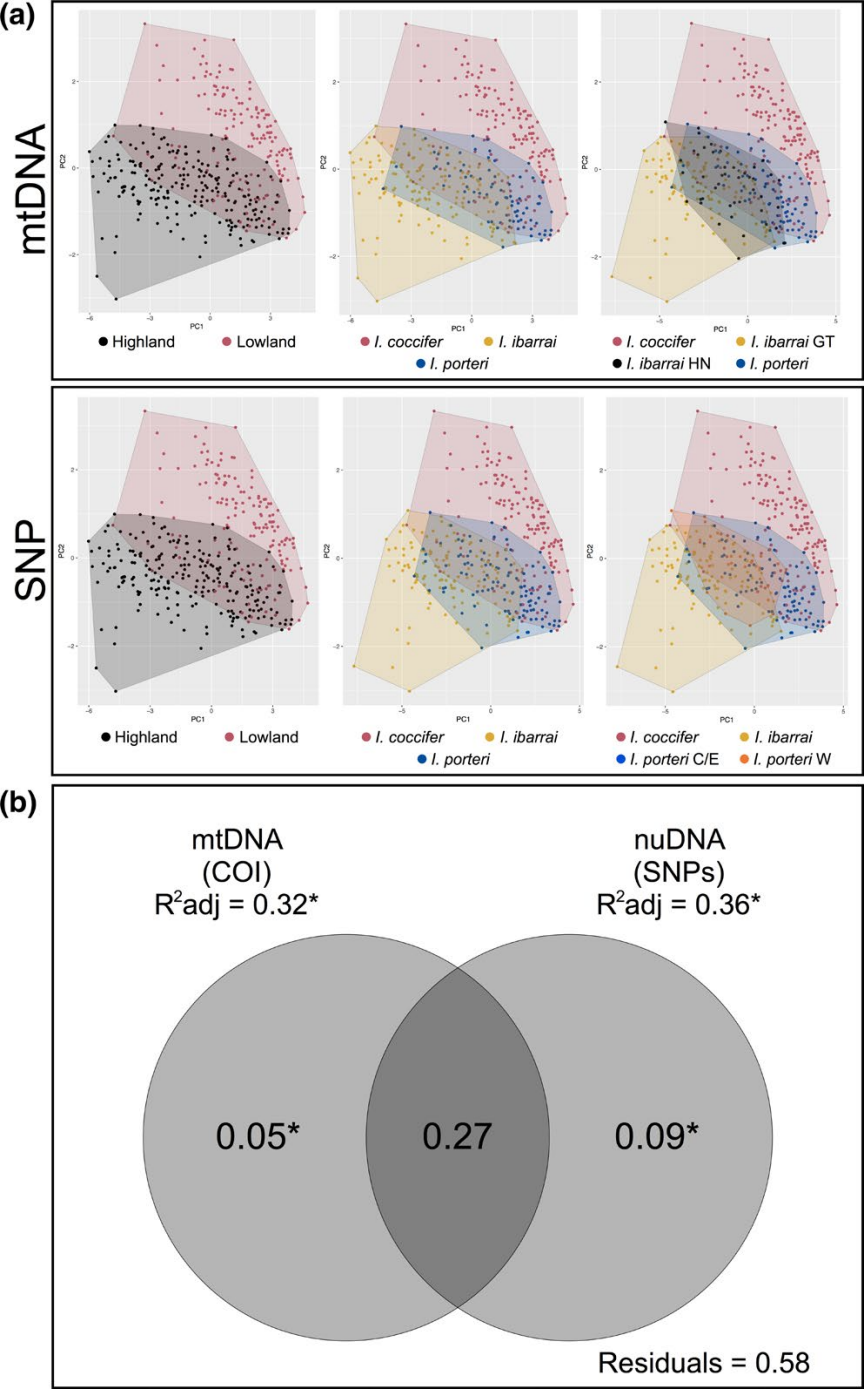
Analyses of the association between species delimitation and morphometry. (a) Visualization of the first two PCA loadings of the morphological variation. Convex polygons were produced using the mtDNA and SNP species assignments for across our molecular species delimitation hypotheses to show the degree of morphological variation and overlap between the species. (b) Venn diagram of the results of the VP analysis that depicts the adjusted *R* ^2^ values and significance levels of the mtDNA‐ and SNP‐based delimitations for discordant highland populations (*I. ibarrai* and *I. porteri*). Values outside of the shaded areas represent marginal effects (*R* ^2^ adj; e.g., the amount of variation explained when testing each delimitation separately). Values in the intersection of the shaded area represent variation explained in common by both delimitations. Values outside of the intersection of the shaded area represent the conditional effects (variation uniquely explained by each delimitation). *p <.001

The morphological variation across the species complex best supports the two species hypothesis, with an overall correct classification rate of 92.4% for both mtDNA and SNP assignments. The three species and four species hypotheses did have high classification rates but were 10.1%–12% lower than the DFAs using a two species classification (Table 1). We found that *I. coccifer* is a morphologically distinct taxon, which had a correct classification rate of 94.9% across all six of the DFAs and showed a high degree of separation in the PCAs (Table 1; Figure 2a). The decreased rate of correct classification in the three and four species DFAs was associated with delineating the Highland taxon into multiple species (Table 1). These populations, which are currently recognized as *I. porteri* and *I. ibarrai*, show a high degree of morphological similarity. Specifically, it is the Honduran populations that are very similar.

The VP analysis found that the nuDNA and mtDNA assignments both significantly explained the dataset (Figure 2b). The nuDNA and mtDNA assignments explained 36% and 32% of the morphological variation, respectively. The VP analysis revealed that 27% of the explained variation was shared by both assignments (Figure 2b). The nuDNA and mtDNA assignments significantly explained 9% (*p* < .001) and 5% (*p* < .001) of the variation as conditional effects, respectively (Figure 2b).

### 3.3 Ecological niche modeling and niche overlap

**FIGURE 3:**
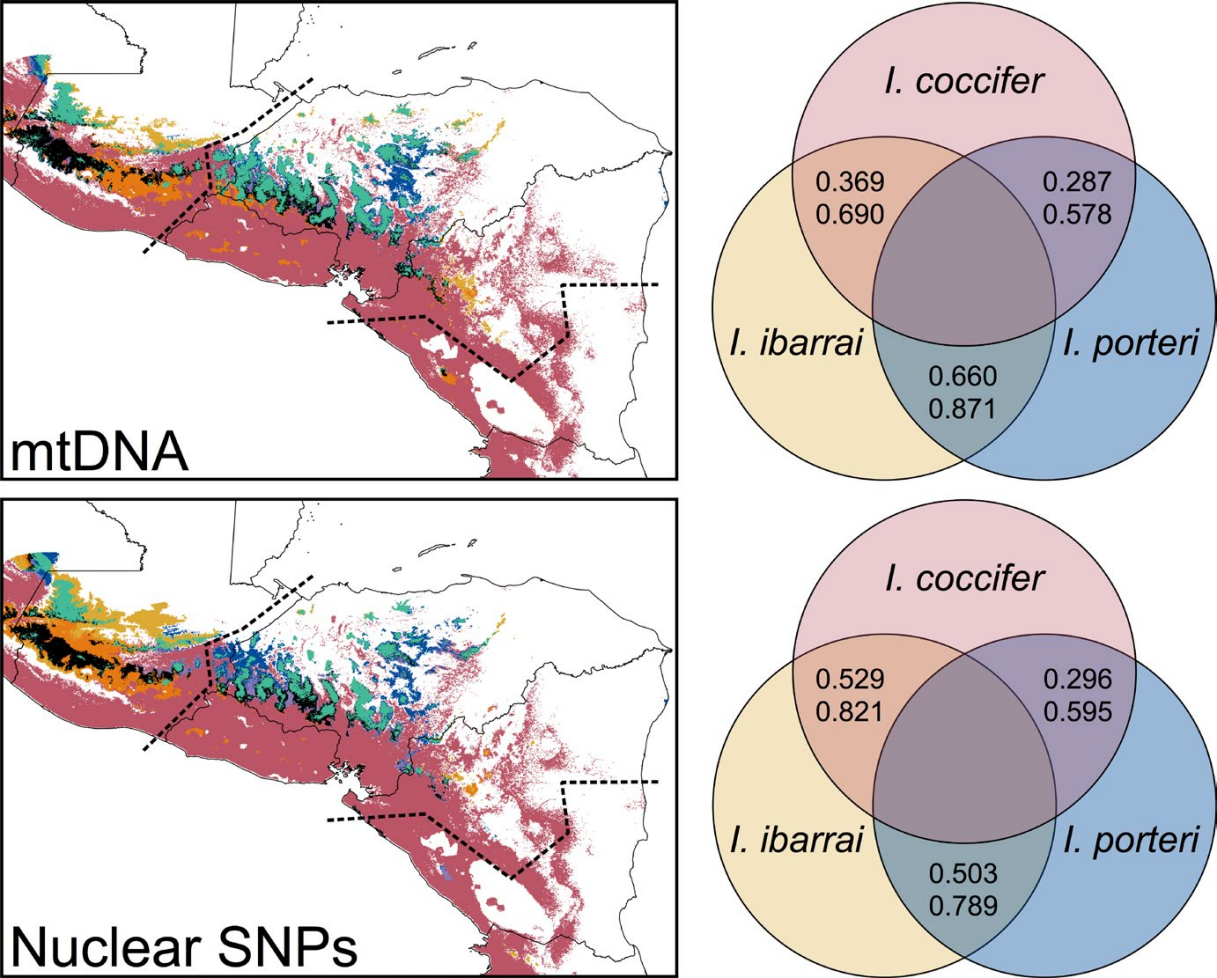
Combined binary maps for the modeled occurrence of the focal taxa based on genetic species assignments. Colors correspond as follows: *I*. *coccifer* only = red; *I. ibarrai* only = yellow; *I. porteri* only = blue; *I. coccifer* + I. *ibarrai* = orange; *I. coccifer* + I. *porteri* = purple; *I. ibarrai* + I. *porteri* = teal; all species present = black; none present = white. Dotted lines represent the boundaries of the Chortís Block. Intersections of focal taxa in the Venn diagrams include the results of niche overlap (Schoener's *D*: top number) and niche identity (*I*: bottom number) tests

The top candidate SDMs that constituted our final models had high AUC scores ranging from 0.81 to 0.95. Based on the known geographic distributions of the species under either phylogenetic hypothesis, very little geographic overestimation occurred in the models. The only model that we see some overestimation into more lowland regions is the *I*. *ibarrai* SNP model (Figure S4), which we attribute to the low number of samples (<20) that have been used for that model. It should be noted that there is evident estimation of the *I. ibarrai* and *I. porteri* models of both datasets (Figure S4) outside of the biogeographic barriers that may delineate the true distributions of these species, which is due to the fact that maxent does not account for physical biogeographic barriers. Binary maps indicating the modeled occurrence (Figure 3) revealed very little distributional overlap between *I. coccifer* and *I. ibarrai* or *I. porteri*. We see more overlap between the *I. coccifer* and *I. ibarrai* within the SNP distribution models, which is primarily due to the overestimation of the *I. ibarrai* SNP model into the lowland areas. Our models did reveal broad zones of overlap between *I. ibarrai* and *I. porteri* under both scenarios, though we see less overlap in the SNP models (Figure 3). The niche overlap and identity tests reflected these results (Figure 3).

## 4 DISCUSSION

Delimiting species can be hindered by a number of factors including highly conserved morphologies (e.g., cryptic species), differences in criteria of species concepts, lineages being in the early stages of the speciation or divergence process, and discordance between gene tree topologies (e.g., mitonuclear discordance). Here we provided an example of a taxonomically confounding species complex of toads from Central America that exhibits extensive mitonuclear discordance and presented an integrative hypothesis testing framework to assess differentiation of integrated data using the different phylogenetic hypotheses caused by mitonuclear discordance. Below we summarize our findings and evidence for the taxonomic relationships of the species within the *I. coccifer* complex, along with a discussion of the conservation implications for this species complex.

### 4.1 Species delimitation

While strides have been made in species delimitation using genetic data (Fujita et al., 2012; Leaché et al., 2018; Luo et al., 2018), taxonomists are still required to make subjective judgments based on multiple separate lines of evidence to decide on the boundary between populations and species (Sites & Marshall, 2004). Following single lines of evidence, such as a single genetic locus, can lead to discordant results that do not reflect an accurate taxonomy (Jackson et al., 2017). We chose to implement single locus species delimitation methods on our mtDNA dataset, and they suggest that there are between 3 and 5 species within the complex. Our tree‐based single locus methods (GMYC and PTP) delineate a species boundary between *I. ibarrai* populations in Guatemala and those in Honduras, which have been previously suggested as two potentially distinct species (Firneno & Townsend, 2019; Mendelson et al., 2011). This is also interesting because the populations of *I. ibarrai* in Honduras correspond to different populations of *I. porteri* according to the SNP dataset. Conversely, ABGD delimits three species units overall, lumping all *I. ibarrai* populations together, and follows the currently applied taxonomy of this complex.

Single locus species delimitation methods rely on the topologies inferred from a single locus and assume that it represents the current species relationship. However, incorrect topologies due to introgression and/or ILS can mislead these methods (Dupuis et al., 2012; Knowles & Carstens, 2007). Because ILS was found to be the cause of mitonuclear discordance in the *I. coccifer* complex and that nuclear introgression was present within populations of *I. porteri* within the Chortís Highlands (Firneno et al., 2020), we also used species delimitation methods that incorporate multilocus data in both a MSC and PSM framework. These methods have been shown to take into account ILS and are robust to low levels of introgression (Zhang et al., 2011). Our multilocus delimitation methods widely varied estimating 1–4 species units. The methods that apply the MSC (BFD* and BPP) tended to delimit species along the lines of our population inferences, whereas our other methods (*gdi* and delineate) were more conservative in their estimates, indicating only one or two species units present. An often‐cited issue with species delimitation methods that use the MSC framework (BFD* and BPP) is that they tend to delimit species boundaries based on population structure, therefore overestimating the number of species within a focal group (Sukumaran & Knowles, 2017). We see this in our data and is the reason why we used comparative methods that implement other frameworks other than the MSC. It has been remarked that the use of *gdi* can have a number of weaknesses including that the criterion depends on the population divergence time relative to the population size (small populations and recent divergence times may skew results), that ambiguity may arise when two populations being compared are of very different sizes, and that the metric has a wide range of indecision and reflects a more subjective nature of species delimitation that these methods are trying to avoid (Leaché et al., 2019). Likewise, it has been argued that the PSM implemented by delineate is unrealistic because it models instantaneous speciation in a single generation, which does not seem to reflect how natural species convert from being an incipient species to a true species (Leaché et al., 2019; Sukumaran & Knowles, 2017). These may be reasons why our heuristic and PSM‐based analyses yielded such conservative results.

The addition of more genetic markers can often aid in the clarity of delimiting species. However, there are cases, as exemplified here, where adding more genetic data can reveal more complexity within and between species than originally assumed or hinder the ability of taxonomists to make objective decisions concerning the delimitation of species (Hinojosa et al., 2019; Ivanov et al., 2018; Papakostas et al., 2016; Pedraza‐Marrón et al., 2019). Due to the discordance between our genetic datasets and the wide array of species units estimated by our species delimitation analyses, it is difficult to discern the structure and number of species in this complex based on genetic data alone. However, we were able to use these competing phylogenetic and delimitation hypotheses to further integrate and evaluate other lines of evidence (e.g., morphology and macroecology) to determine which hypothesis best describes the variation seen in these other data types and most accurately reflects the proper taxonomic relationships and distributions of the species in this complex.

### 4.2 Taxonomic implications

Under the GLC, the only necessary property of a species is its existence as an independently evolving lineage (de Queiroz, 2005, 2007). Following our molecular phylogenetic and species delimitation methodologies under the GLC, we cannot clearly and objectively make an inference of the number of species based on our genetic data. Also, due to mitonuclear discordance it is still not clear which dataset (mtDNA or SNP) gives us a more concrete indication of where the species range limits lie in this complex. Therefore, we integrated morphological and macroecological analyses to further test hypotheses of species number and distribution. These combinations of data in an integrative taxonomic framework have long been shown to clarify problematic species complexes such as the *I. coccifer* complex (Padial et al., 2010). It should be noted that, though macroecological modeling is not often used for species delimitation, when combined with genetic and/or morphological information, species delimitation hypotheses can improve in objectivity and strength (Hidalgo‐Galiana et al., 2014; Leaché et al., 2009; Razkin et al., 2016; Rissler & Apodaca, 2007).

Though Firneno et al. (2020) suggest that there may be more than three species in this complex, a hypothesis that was based only on genetic data, we do not find evidence within our genetic, morphological, and macroecological data for this claim. Within our morphological data, we see little separation between highland populations within Honduras (Figure 2a) and a low amount of support for discerning these populations (Table 1) when they are treated as separate species; however, we do see good support for a separate highland population in Guatemala. Instead, we see high support for only two species (a lowland entity and a highland entity) and moderate support for three species (a lowland entity and two highland entities) with relatively high overlap between the two highland entities. Our macroecological analyses clearly show separation of suitable habitat between at least two species (Figure 3), but with higher niche overlap when splitting highland entities into two species/populations. It is also worth noting that, while we could not test a four species hypothesis within our macroecological analyses (the locality datasets upon spatial rarefication become too small to generate reliable models), we would expect high levels of niche overlap as we see in our current three species models (Figure 3).

Synthesizing the contrasting datasets that we have collected and analyzed, it is still difficult to concretely say how many species there are in this complex. Though we can safely rule out hypotheses concerning four or more species in this complex, it seems as if there is sufficient evidence for two or three species. We suggest that there are three species in this complex under the GLC based on the following criteria: (a) There are at least three well‐supported monophyletic lineages within our phylogenies, though the mtDNA relationship is rendered paraphyletic when compared to the SNP phylogeny due to mitonuclear discordance); (b) there are four distinct populations that reflect the lineages from the SNP dataset, though there is gene flow occurring between two of the population lineages indicating a possible lack of reproductive isolation; (c) there is support for three morphologically distinct entities that reflect our phylogenetic lineages with a high degree of morphological conservation between them; and (d) there is differentiation between three macroecological entities that reflect out phylogenetic lineages, though there is a moderate to high degree of niche overlap between highland lineages.

Along with the species limits, we were also concerned with determining which distribution (mtDNA or SNP) reflected the taxonomy as well. We found support for a more parsimonious distributional hypothesis based on the SNP dataset based on complete monophyly among all of our lineages phylogenetically (Figure 1e), better differentiation among morphological entities under the SNP hypothesis (Figure 2b; Table 1), and less niche overlap between highland species (Figure 3). The distributions include (a) *I. coccifer*, which comprises all lowland populations within and outside of the Chortís Block; (b) *I. ibarrai*, which comprises all highland populations west of the Chortís Highlands in Guatemala; and (c) *I. porteri*, which comprises all highland populations within the Chortís Highlands. These distributions make sense not only based on the genetic, morphological, and macroecological data that we present, but also the biogeographic barriers that divide these species distributions. *I. coccifer* is clearly separate from the two highland taxa by a highland barrier, and *I. ibarrai* and *I. porteri* are separated from each other by the Motagua‐Polochic fault system at the western border of the Chortís Block, a well‐supported biogeographic boundary for amphibians (Figures 1b and S5) (Crawford & Smith, 2005; Mendoza‐Henao et al., 2020; Rovito et al., 2015). It may also be worth noting that *I. porteri's* distribution may extend into the highlands of Nicaragua (still comprising the Chortís Highlands), due to the lack of any biogeographic separation. However, no confirmed samples have ever been reported from this region.

Ultimately, we were able to use integrated lines of evidence in a hypothesis testing framework to evaluate the species limits and distributions of the three species found within this highly confounding species complex. A major factor that may also be confounding this species complex is that it is a relatively young complex (divergence was estimated at ~790,000 years ago; Firneno et al. (2020). Young lineages may still be undergoing the process of speciation/divergence, which can prove challenging for the types of delimitation methods that we bring to bear (Fujita et al., 2012; Padial et al., 2010). In the future, more taxon‐specific lines of evidence (e.g. bioacoustics, osteology, etc.) may be useful in clarifying and solidifying this complex’s taxonomy further, as well as using a process‐based delimitation approach that integrates processes of speciation into delimitation practices (Smith & Carstens, 2020). Finally, due to the long‐standing taxonomy and distributions that were originally proposed by Mendelson et al. (2005), there have been existing conservation recommendations that may now need to be reevaluated based upon these new findings.

### 4.3 Conservation implications

A stable (Smith & Carstens, 2020) taxonomy is necessary to guide conservation assessment measures and practices for species. Central American amphibians, such as those within the *I. coccifer* complex, represent some of the world's most vulnerable organisms due to rapid habitat loss and zoonotic disease (e.g., chytridiomycosis) in this region (Gallant et al., 2007; James et al., 2015; Lips, 1998, 2016; Townsend et al., 2010; Whitfield et al., 2016); therefore, stable taxonomies are necessary for proper conservation. A recent IUCN reevaluation of this complex assessed the distribution of *I. porteri* on the guidance of Firneno et al. (2020) and changed its conservation status from Data Deficient to Least Concern; currently, the IUCN recognizes *I. coccifer* and *I porteri* as Least Concern and *I. ibarrai* as Endangered. However, the distribution and conservation of *I. ibarrai* are awaiting reevaluation in the IUCN Red List, and we propose that the distribution of *I. ibarrai* should be restricted to everything west of the Chortís/Guatemalan Highlands boundary (Figures 1b and S5), and we suggest that *I. ibarrai's* conservation status be reevaluated to accurately reflect this restricted range distribution.

While we sought to evaluate the species limits of the *Incilius coccifer* complex, we also wanted to provide an integrative hypothesis testing framework to aid in the delimitation of species complexes in the face of mitonuclear discordance. Our results highlight the importance of using comprehensive and integrated data for addressing complex species delimitation problems. Our findings further emphasize the necessity of an accurate taxonomy to guide conservation measures, especially among organisms that inhabit ecosystems that are highly imperiled. With this integrative taxonomic approach, we hope to fuel a novel perspective on pursuing species delimitation and phylogeographic work using complex and cryptic species and work within this region of Central America where amphibians (and other organisms) are facing grave conservation concerns due to anthropogenic changes to their ecosystems.

## CONFLICT OF INTEREST

We declare that we have no competing interests.

## AUTHOR CONTRIBUTION

**Thomas Firneno:** Conceptualization (lead); Data curation (lead); Formal analysis (lead); Funding acquisition (equal); Investigation (lead); Methodology (equal); Project administration (lead); Resources (lead); Software (equal); Supervision (lead); Validation (equal); Visualization (lead); Writing‐original draft (lead); Writing‐review & editing (equal). **Justin R O'Neill:** Data curation (supporting); Formal analysis (equal); Investigation (equal); Methodology (equal); Project administration (equal); Validation (equal); Visualization (equal); Writing‐original draft (supporting); Writing‐review & editing (equal). **Michael W Itgen:** Data curation (supporting); Formal analysis (equal); Investigation (equal); Methodology (equal); Validation (equal); Visualization (equal); Writing‐original draft (supporting); Writing‐review & editing (equal). **Timothy A Kihneman:** Data curation (supporting); Formal analysis (supporting); Investigation (supporting); Methodology (supporting); Validation (supporting); Visualization (supporting); Writing‐review & editing (supporting). **Josiah H Townsend:** Conceptualization (supporting); Validation (supporting); Writing‐review & editing (supporting). **Matthew K. Fujita:** Conceptualization (supporting); Funding acquisition (supporting); Investigation (supporting); Methodology (supporting); Resources (supporting); Writing‐original draft (supporting); Writing‐review & editing (supporting).

## Supporting information

SupInfo S1

## Data Availability

We have included a large data package on DRYAD (https://doi.org/10.5061/dryad.j9kd51cb5) that includes our final ddRADseq filtered “haplotypes” files, COI alignment files, resulting input files for a number of analysis programs (structure, admixture, DAPC, fineradstructure, BFD*, BPP, gdi, delineate, beast, and snapp), an excel spreadsheet of raw morphological measurements split into the two distributional datasets used, and a.csv file of both locality datasets used for SDMs.
