# Supplementary material for: Delimitation despite discordance: Evaluating the species limits of a confounding species complex in the face of mitonuclear discordance
Source: Ecol Evol. 2021 Aug 11;11(18):12739–53. doi: 10.1002/ece3.8018 (PMC8462145; doi:10.1002/ece3.8018)
Supplement: Supplementary file 1 — SupInfo S1 [file ECE3-11-12739-s001.docx]

**Supplementary information for:**

**Delimitation despite discordance: evaluating the species limits of a confounding species complex in the face of mitonuclear discordance**

THOMAS J. FIRNENO, JR.^1,2,^*, JUSTIN R. O’NEILL^3^, MICHAEL W. ITGEN^4^, TIMOTHY A. KIHNEMAN^1^, JOSIAH H. TOWNSEND^5,6^, & MATTHEW K. FUJITA^1,2^

^1^ *Department of Biology, University of Texas at Arlington, Arlington, Texas, 76019, USA*

^2^ *Amphibian and Reptile Diversity Research Center, Department of Biology, University of Texas at Arlington, Arlington, Texas, 76019, USA*

^3^ *Department of Biology, University of Maryland, College Park, MD, 20742, MD, USA*

^4^ *Department of Biology, Colorado State University, Fort Collins, CO 80523, USA*

^5^ *Department of Biology, Indiana University of Pennsylvania, Indiana, Pennsylvania 15705, USA*

^6^ *Centro Zamorano de Biodiversidad, Departamento de Ambiente y Desarrollo, Escuela Agrícola Panamericana Zamorano, Municipalidad de San Antonio de Oriente, Francisco Morazán, Honduras*

* corresponding author: thomas.firneno@uta.edu

**Table S1.** Samples used for molecular data analyses, with locality data, lineage specificity (both mtDNA and nDNA), voucher numbers, and GenBank Accession numbers; GT, Guatemala; HN, Honduras; MX, Mexico; NI, Nicaragua; PA, Panama.

| mtDNA Lineage | SNP Lineage | Field  Number | Museum Voucher  Number | Locality | GenBank: mtDNA |
| --- | --- | --- | --- | --- | --- |
| *I. coccifer* | *I. coccifer* | JS1150 | — | NI: Región Autónoma Atlántica Norte | MK720880 |
| *I. coccifer* | *I. coccifer* | JS1058 | — | NI: Rivas: Ometepe | MK720881 |
| *I. coccifer* | *I. coccifer* | JS1016 | — | NI: Rivas: Ometepe | MK720883 |
| *I. coccifer* | *I. coccifer* | JHT3301 | USNM 578693 | HN: Valle: Isla el Tigre | KR736042 |
| *I. porteri* | *I. coccifer* | JHT3302 | USNM 578694 | HN: Valle: Isla el Tigre | KR736043 |
| *I. ibarrai* | *I. ibarrai* | JAC19612 | UTA A-52528 | GT: Quiché | JN867971 |
| — | *I. ibarrai* | ENS13348 | — | GT: Santa Rosa: Carretera Ayarza | — |
| — | *I. ibarrai* | ENS13349 | — | GT: Santa Rosa: Carretera Ayarza | — |
| *I. porteri* | *I. porteri* | CAC044 | USNM 578695 | HN: Comayagua: Cerro Zarciadero | MK720846 |
| *I. ibarrai* | *I. porteri* | IRL002 | — | HN: Comayagua: Cerro Zarciadero | MK720865 |
| — | *I. porteri* | IRL003 | — | HN: Comayagua: Cerro Zarciadero | — |
| *I. ibarrai* | *I. porteri* | IRL005 | — | HN: Comayagua: Cerro Zarciadero | MK720866 |
| — | *I. porteri* | IRL006 | — | HN: Comayagua: Cerro Zarciadero | — |
| *I. porteri* | *I. porteri* | IRL010 | — | HN: Comayagua: Cerro Zarciadero | MK720838 |
| *I. porteri* | *I. porteri* | IRL011 | — | HN: Comayagua: Cerro Zarciadero | MK720849 |
| *I. porteri* | *I. porteri* | JHT2149 | — | HN: Francisco Morazán: Uyuca | MK720847 |
| *I. ibarrai* | *I. porteri* | JHT2205 | — | HN: Comayagua: Cerro Zarciadero | MK720863 |
| — | *I. porteri* | JHT2206 | — | HN: Comayagua: Cerro Zarciadero | — |
| *I. porteri* | *I. porteri* | JHT2228 | — | HN: Francisco Morazán: Finca la Alondra | MK720843 |
| *I. porteri* | *I. porteri* | JHT2246 | — | HN: Francisco Morazán: Uyuca | MK720839 |
| *I. porteri* | *I. porteri* | JHT2256 | — | HN: Francisco Morazán: Uyuca | MK720844 |
| *I. porteri* | *I. porteri* | JHT2257 | — | HN: Francisco Morazán: Uyuca | MK720841 |
| *I. porteri* | *I. porteri* | JHT3947 | CM 163380 | HN: Francisco Morazán: Uyuca | MT348746 |
| *I. ibarrai* | *I. porteri* | JHT2604 | — | HN: La Paz: Guajiquiro | MK720864 |
| *I. ibarrai* | *I. porteri* | JHT2605 | — | HN: La Paz: Guajiquiro | MK720860 |
| *I. ibarrai* | *I. porteri* | JHT2608 | — | HN: La Paz: Guajiquiro | MK720853 |
| *I. ibarrai* | *I. porteri* | JHT2609 | — | HN: La Paz: Guajiquiro | MK720874 |
| *I. porteri* | *I. porteri* | JHT2610 | — | HN: La Paz: Guajiquiro | MK720837 |
| — | *I. porteri* | JHT2611 | — | HN: La Paz: Guajiquiro | — |
| *I. ibarrai* | *I. porteri* | JHT2612 | — | HN: La Paz: Guajiquiro | MK720871 |
| *I. ibarrai* | *I. porteri* | JHT2613 | — | HN: La Paz: Guajiquiro | MK720862 |
| *I. ibarrai* | *I. porteri* | JHT2615 | — | HN: La Paz: Guajiquiro | MK720855 |
| — | *I. porteri* | JHT2616 | — | HN: La Paz: Guajiquiro | — |
| *I. ibarrai* | *I. porteri* | JHT2906 | — | HN: Intibucá: El Rodeo | MK720852 |
| *I. ibarrai* | *I. porteri* | JHT3724 | CM 163392 | HN: Intibucá: El Rodeo | MT348755 |
| *I. ibarrai* | *I. porteri* | JHT3725 | CM 163393 | HN: Intibucá: El Rodeo | MT348756 |
| *I. ibarrai* | *I. porteri* | JHT3726 | CM 163394 | HN: Intibucá: El Rodeo | MT348757 |
| *I. ibarrai* | *I. porteri* | JHT3728 | CM 163396 | HN: Intibucá: El Rodeo | MT348758 |
| *I. ibarrai* | *I. porteri* | JHT3729 | CM 163397 | HN: Intibucá: El Rodeo | MT348759 |
| *I. ibarrai* | *I. porteri* | JHT3730 | CM 163398 | HN: Intibucá: El Rodeo | MT348760 |
| *I. ibarrai* | *I. porteri* | JHT3731 | CM 163399 | HN: Intibucá: El Rodeo | MT348761 |
| *I. ibarrai* | *I. porteri* | JHT3732 | CM 163400 | HN: Intibucá: El Rodeo | MT348762 |
| *I. ibarrai* | *I. porteri* | JHT3733 | CM 168168 | HN: Intibucá: El Rodeo | MK720875 |
| *I. ibarrai* | *I. porteri* | JHT3777 | CM 168169 | HN: Intibucá: El Rodeo | MT348763 |
| *I. coccifer* | *I. porteri* | JHT3793 | CM 168171 | HN: Intibucá: San Pedro la Loma | MK720884 |
| *I. ibarrai* | *I. porteri* | JHT3794 | CM 168172 | HN: Intibucá: San Pedro la Loma | MK720872 |
| *I. ibarrai* | *I. porteri* | JHT3795 | CM 163306 | HN: Intibucá: San Pedro la Loma | MT348764 |
| *I. ibarrai* | *I. porteri* | JHT3922 | CM 168174 | HN: Intibucá: El Rodeo | MT348765 |
| *I. ibarrai* | *I. porteri* | JHT3923 | CM 168175 | HN: Intibucá: El Rodeo | MT348766 |
| *I. ibarrai* | *I. porteri* | JHT3924 | CM 168176 | HN: Intibucá: San Pedro la Loma | MT348767 |
| *I. porteri* | *I. porteri* | JHT3925 | CM 168179 | HN: Intibucá: Opalaca | MK720840 |
| *I. porteri* | *I. porteri* | JHT3932 | CM 168186 | HN: Intibucá: El Rodeo | MT348745 |
| *I. ibarrai* | *I. porteri* | ENS10270 | UTA A-53662 | HN: Ocotepeque | JN867970 |
| *I. ibarrai* | *I. porteri* | JHT3692 | CM 163384 | HN: Lempira: La Ventanas | MT348748 |
| *I. ibarrai* | *I. porteri* | JHT3694 | CM 163385 | HN: Lempira: La Ventanas | MT348747 |
| *I. ibarrai* | *I. porteri* | JHT3696 | CM 163386 | HN: Lempira: La Ventanas | MK720873 |
| *I. ibarrai* | *I. porteri* | JHT3697 | CM 163387 | HN: Lempira: La Ventanas | MT348749 |
| *I. ibarrai* | *I. porteri* | JHT3698 | CM 163388 | HN: Lempira: La Ventanas | MT348750 |
| *I. ibarrai* | *I. porteri* | JHT3699 | CM 163389 | HN: Lempira: La Ventanas | MT348751 |
| *I. ibarrai* | *I. porteri* | JHT3700 | CM 163390 | HN: Lempira: La Ventanas | MT348752 |
| *I. ibarrai* | *I. porteri* | JHT3782 | CM 168170 | HN: Lempira: La Ventanas | MT348753 |
| *I. ibarrai* | *I. porteri* | JHT3921 | CM 168173 | HN: Lempira: La Ventanas | MK720869 |
| *I. ibarrai* | *I. porteri* | JHT3945 | CM 168177 | HN: Lempira: La Ventanas | MK720876 |
| *I. ibarrai* | *I. porteri* | JHT3946 | CM 168178 | HN: Lempira: La Ventanas | MT348754 |
| *I. cycladen* | — | JRM4607 | UTA A-54847 | MX: Guerrero: Agua de Obispo | JN867967 |
| *I. signifer* | — | JRM 4968 | UTA A-JRM 4968 | PA: Cocle: El Copé | JN867988 |

**Table S2.** Bayes Factor Delimitation results for each model analysis. The number of species represents the number of species included in each analysis after lumping or splitting lineages.

| Model | Species | MLE | BF | Rank |
| --- | --- | --- | --- | --- |
|  |  |  |  |  |
| Current Taxonomy | 3 | -16377.806 | — | 2 |
| *I. coccifer*, *I. ibarrai*, and *I. porteri* |  |  |  |  |
| Lump Highland | 2 | -19958.855 | 7162.098 | 3 |
| *I. coccifer*, *I. ibarrai* + *I. porteri* |  |  |  |  |
| Split All Populations | 4 | -15893.671 | -484.135 | 1 |
| *I. coccifer*, *I. ibarrai*, *I. porteri* West, and *I. porteri* East | | |  |  |
| Split All, Clump Some Highlands | 3 | -19253.439 | 5751.266 | 4 |
| *I. coccifer*, *I. ibarrai* + *I. porteri* West, and *I. porteri* East | |  |  |  |

**Table S3.** Genetically verified (from this and previously published data) and VertNet search results locality data used for species distribution models and the species assignments of occurrence records based on discordant genetic datasets.

| Locality | | Genetic Assignment | |
| --- | --- | --- | --- |
| Latitude | Longitude | mtDNA | SNP |
| 9.9167 | -84.1333 | *I. coccifer* | *I. coccifer* |
| 9.952109 | -84.373238 | *I. coccifer* | *I. coccifer* |
| 9.981572 | -84.746237 | *I. coccifer* | *I. coccifer* |
| 9.927956 | -84.067083 | *I. coccifer* | *I. coccifer* |
| 9.909131 | -84.135021 | *I. coccifer* | *I. coccifer* |
| 10.026993 | -84.723326 | *I. coccifer* | *I. coccifer* |
| 14.0904 | -89.07446 | *I. coccifer* | *I. coccifer* |
| 14.0904 | -89.07446 | *I. coccifer* | *I. coccifer* |
| 14.0904 | -89.07446 | *I. coccifer* | *I. coccifer* |
| 14.0904 | -89.07446 | *I. coccifer* | *I. coccifer* |
| 14.36667 | -89.1 | *I. coccifer* | *I. coccifer* |
| 14.36667 | -89.1 | *I. coccifer* | *I. coccifer* |
| 14.36667 | -89.1 | *I. coccifer* | *I. coccifer* |
| 14.36667 | -89.1 | *I. coccifer* | *I. coccifer* |
| 14.36667 | -89.1 | *I. coccifer* | *I. coccifer* |
| 14.36667 | -89.1 | *I. coccifer* | *I. coccifer* |
| 14.36667 | -89.1 | *I. coccifer* | *I. coccifer* |
| 13.71667 | -89.00729 | *I. coccifer* | *I. coccifer* |
| 13.71667 | -89.00729 | *I. coccifer* | *I. coccifer* |
| 13.71667 | -89.00729 | *I. coccifer* | *I. coccifer* |
| 13.71667 | -89.00729 | *I. coccifer* | *I. coccifer* |
| 13.71667 | -89.00729 | *I. coccifer* | *I. coccifer* |
| 13.71667 | -89.00729 | *I. coccifer* | *I. coccifer* |
| 13.71667 | -88.93333 | *I. coccifer* | *I. coccifer* |
| 13.71667 | -88.93333 | *I. coccifer* | *I. coccifer* |
| 13.71667 | -88.93333 | *I. coccifer* | *I. coccifer* |
| 13.71667 | -88.93333 | *I. coccifer* | *I. coccifer* |
| 13.71667 | -88.93333 | *I. coccifer* | *I. coccifer* |
| 13.71152 | -88.92807 | *I. coccifer* | *I. coccifer* |
| 13.71152 | -88.92807 | *I. coccifer* | *I. coccifer* |
| 13.71667 | -88.91484 | *I. coccifer* | *I. coccifer* |
| 13.78619 | -89.38853 | *I. coccifer* | *I. coccifer* |
| 13.78619 | -89.38853 | *I. coccifer* | *I. coccifer* |
| 13.78619 | -89.38853 | *I. coccifer* | *I. coccifer* |
| 13.78619 | -89.38853 | *I. coccifer* | *I. coccifer* |
| 13.78619 | -89.38853 | *I. coccifer* | *I. coccifer* |
| 13.78619 | -89.38853 | *I. coccifer* | *I. coccifer* |
| 13.78619 | -89.38853 | *I. coccifer* | *I. coccifer* |
| 13.78619 | -89.38853 | *I. coccifer* | *I. coccifer* |
| 13.78619 | -89.38853 | *I. coccifer* | *I. coccifer* |
| 13.78619 | -89.38853 | *I. coccifer* | *I. coccifer* |
| 13.78619 | -89.38853 | *I. coccifer* | *I. coccifer* |
| 13.78619 | -89.38853 | *I. coccifer* | *I. coccifer* |
| 13.78619 | -89.38853 | *I. coccifer* | *I. coccifer* |
| 13.78619 | -89.38853 | *I. coccifer* | *I. coccifer* |
| 13.78619 | -89.38853 | *I. coccifer* | *I. coccifer* |
| 13.78619 | -89.38853 | *I. coccifer* | *I. coccifer* |
| 13.78619 | -89.38853 | *I. coccifer* | *I. coccifer* |
| 13.78619 | -89.38853 | *I. coccifer* | *I. coccifer* |
| 13.78619 | -89.38853 | *I. coccifer* | *I. coccifer* |
| 13.7 | -89.40772 | *I. coccifer* | *I. coccifer* |
| 13.7 | -89.40772 | *I. coccifer* | *I. coccifer* |
| 13.7 | -89.40772 | *I. coccifer* | *I. coccifer* |
| 13.7 | -89.40772 | *I. coccifer* | *I. coccifer* |
| 13.7 | -89.40772 | *I. coccifer* | *I. coccifer* |
| 13.7 | -89.40772 | *I. coccifer* | *I. coccifer* |
| 13.7 | -89.40772 | *I. coccifer* | *I. coccifer* |
| 13.7 | -89.40772 | *I. coccifer* | *I. coccifer* |
| 13.7 | -89.40772 | *I. coccifer* | *I. coccifer* |
| 13.7 | -89.40772 | *I. coccifer* | *I. coccifer* |
| 13.7 | -89.40772 | *I. coccifer* | *I. coccifer* |
| 13.7 | -89.40772 | *I. coccifer* | *I. coccifer* |
| 13.7 | -89.40772 | *I. coccifer* | *I. coccifer* |
| 13.7 | -89.40772 | *I. coccifer* | *I. coccifer* |
| 13.95 | -88.16667 | *I. coccifer* | *I. coccifer* |
| 13.71029 | -89.21052 | *I. coccifer* | *I. coccifer* |
| 13.71029 | -89.21052 | *I. coccifer* | *I. coccifer* |
| 13.71029 | -89.21052 | *I. coccifer* | *I. coccifer* |
| 13.71029 | -89.21052 | *I. coccifer* | *I. coccifer* |
| 13.71029 | -89.21052 | *I. coccifer* | *I. coccifer* |
| 13.71029 | -89.21052 | *I. coccifer* | *I. coccifer* |
| 13.71029 | -89.21052 | *I. coccifer* | *I. coccifer* |
| 13.71029 | -89.21052 | *I. coccifer* | *I. coccifer* |
| 13.71029 | -89.21052 | *I. coccifer* | *I. coccifer* |
| 13.71029 | -89.21052 | *I. coccifer* | *I. coccifer* |
| 13.71029 | -89.21052 | *I. coccifer* | *I. coccifer* |
| 13.71029 | -89.21052 | *I. coccifer* | *I. coccifer* |
| 13.7 | -89.2 | *I. coccifer* | *I. coccifer* |
| 13.7 | -89.2 | *I. coccifer* | *I. coccifer* |
| 13.7 | -89.2 | *I. coccifer* | *I. coccifer* |
| 13.7 | -89.2 | *I. coccifer* | *I. coccifer* |
| 13.7 | -89.05209 | *I. coccifer* | *I. coccifer* |
| 13.66667 | -89.1 | *I. coccifer* | *I. coccifer* |
| 13.66667 | -89.1 | *I. coccifer* | *I. coccifer* |
| 13.66667 | -89.1 | *I. coccifer* | *I. coccifer* |
| 13.66667 | -89.1 | *I. coccifer* | *I. coccifer* |
| 13.66667 | -89.1 | *I. coccifer* | *I. coccifer* |
| 13.66667 | -89.1 | *I. coccifer* | *I. coccifer* |
| 13.66667 | -89.1 | *I. coccifer* | *I. coccifer* |
| 13.66667 | -89.1 | *I. coccifer* | *I. coccifer* |
| 13.48333 | -89.22431 | *I. coccifer* | *I. coccifer* |
| 13.48333 | -89.22431 | *I. coccifer* | *I. coccifer* |
| 13.48333 | -89.22431 | *I. coccifer* | *I. coccifer* |
| 13.48333 | -89.22431 | *I. coccifer* | *I. coccifer* |
| 13.48333 | -89.22431 | *I. coccifer* | *I. coccifer* |
| 13.48333 | -89.22431 | *I. coccifer* | *I. coccifer* |
| 13.48333 | -89.22431 | *I. coccifer* | *I. coccifer* |
| 13.48333 | -89.22431 | *I. coccifer* | *I. coccifer* |
| 13.48333 | -89.22431 | *I. coccifer* | *I. coccifer* |
| 13.48333 | -89.22431 | *I. coccifer* | *I. coccifer* |
| 13.48333 | -89.22431 | *I. coccifer* | *I. coccifer* |
| 13.48333 | -89.22431 | *I. coccifer* | *I. coccifer* |
| 13.48333 | -89.22431 | *I. coccifer* | *I. coccifer* |
| 13.48333 | -89.22431 | *I. coccifer* | *I. coccifer* |
| 13.48333 | -89.22431 | *I. coccifer* | *I. coccifer* |
| 13.48333 | -89.22431 | *I. coccifer* | *I. coccifer* |
| 13.48333 | -89.22431 | *I. coccifer* | *I. coccifer* |
| 13.48333 | -89.22431 | *I. coccifer* | *I. coccifer* |
| 13.48333 | -89.22431 | *I. coccifer* | *I. coccifer* |
| 13.48333 | -89.22431 | *I. coccifer* | *I. coccifer* |
| 13.48333 | -89.22431 | *I. coccifer* | *I. coccifer* |
| 13.48333 | -89.22431 | *I. coccifer* | *I. coccifer* |
| 13.48333 | -89.22431 | *I. coccifer* | *I. coccifer* |
| 13.48333 | -89.22431 | *I. coccifer* | *I. coccifer* |
| 13.48333 | -89.22431 | *I. coccifer* | *I. coccifer* |
| 13.48333 | -89.22431 | *I. coccifer* | *I. coccifer* |
| 13.48333 | -89.22431 | *I. coccifer* | *I. coccifer* |
| 13.48333 | -89.22431 | *I. coccifer* | *I. coccifer* |
| 13.48333 | -89.22431 | *I. coccifer* | *I. coccifer* |
| 13.48333 | -89.22431 | *I. coccifer* | *I. coccifer* |
| 13.48333 | -89.22431 | *I. coccifer* | *I. coccifer* |
| 13.48333 | -89.22431 | *I. coccifer* | *I. coccifer* |
| 13.48333 | -89.22431 | *I. coccifer* | *I. coccifer* |
| 13.48333 | -89.22431 | *I. coccifer* | *I. coccifer* |
| 13.48333 | -89.22431 | *I. coccifer* | *I. coccifer* |
| 13.48333 | -89.22431 | *I. coccifer* | *I. coccifer* |
| 13.48333 | -89.22431 | *I. coccifer* | *I. coccifer* |
| 13.48333 | -89.22431 | *I. coccifer* | *I. coccifer* |
| 13.48333 | -89.22431 | *I. coccifer* | *I. coccifer* |
| 13.48333 | -89.22431 | *I. coccifer* | *I. coccifer* |
| 13.48333 | -89.22431 | *I. coccifer* | *I. coccifer* |
| 13.48333 | -89.22431 | *I. coccifer* | *I. coccifer* |
| 13.48333 | -89.22431 | *I. coccifer* | *I. coccifer* |
| 13.48333 | -89.22431 | *I. coccifer* | *I. coccifer* |
| 13.48333 | -89.22431 | *I. coccifer* | *I. coccifer* |
| 13.48333 | -89.22431 | *I. coccifer* | *I. coccifer* |
| 13.48333 | -89.22431 | *I. coccifer* | *I. coccifer* |
| 13.7 | -89.2 | *I. coccifer* | *I. coccifer* |
| 13.7 | -89.2 | *I. coccifer* | *I. coccifer* |
| 13.86667 | -89.51667 | *I. coccifer* | *I. coccifer* |
| 13.66667 | -88.85742 | *I. coccifer* | *I. coccifer* |
| 13.66667 | -88.80659 | *I. coccifer* | *I. coccifer* |
| 13.835036 | -88.991881 | *I. coccifer* | *I. coccifer* |
| 13.72114 | -89.00632 | *I. coccifer* | *I. coccifer* |
| 13.637212 | -89.104758 | *I. coccifer* | *I. coccifer* |
| 13.327846 | -88.044912 | *I. coccifer* | *I. coccifer* |
| 13.5391998 | -87.346001 | *I. coccifer* | *I. coccifer* |
| 13.30028 | -87.19083 | *I. coccifer* | *I. coccifer* |
| 13.31465 | -87.19769 | *I. coccifer* | *I. coccifer* |
| 13.31465 | -87.19769 | *I. coccifer* | *I. coccifer* |
| 14.80639 | -85.90657 | *I. coccifer* | *I. coccifer* |
| 14.80639 | -85.90657 | *I. coccifer* | *I. coccifer* |
| 14.80639 | -85.90657 | *I. coccifer* | *I. coccifer* |
| 14.80639 | -85.90657 | *I. coccifer* | *I. coccifer* |
| 14.80639 | -85.90657 | *I. coccifer* | *I. coccifer* |
| 14.80639 | -85.90657 | *I. coccifer* | *I. coccifer* |
| 14.80639 | -85.90657 | *I. coccifer* | *I. coccifer* |
| 14.80639 | -85.90657 | *I. coccifer* | *I. coccifer* |
| 14.8 | -85.9 | *I. coccifer* | *I. coccifer* |
| 15.1 | -85.53333 | *I. coccifer* | *I. coccifer* |
| 13.58333 | -87.57206 | *I. coccifer* | *I. coccifer* |
| 13.58333 | -87.57206 | *I. coccifer* | *I. coccifer* |
| 13.58333 | -87.57206 | *I. coccifer* | *I. coccifer* |
| 13.58333 | -87.57206 | *I. coccifer* | *I. coccifer* |
| 13.58333 | -87.57206 | *I. coccifer* | *I. coccifer* |
| 14.486911 | -89.632368 | *I. coccifer* | *I. coccifer* |
| 14.596426 | -89.455693 | *I. coccifer* | *I. coccifer* |
| 14.596426 | -89.455693 | *I. coccifer* | *I. coccifer* |
| 14.320935 | -91.91243 | *I. coccifer* | *I. coccifer* |
| 14.320935 | -91.91243 | *I. coccifer* | *I. coccifer* |
| 14.320935 | -91.91243 | *I. coccifer* | *I. coccifer* |
| 14.320935 | -91.91243 | *I. coccifer* | *I. coccifer* |
| 14.320935 | -91.91243 | *I. coccifer* | *I. coccifer* |
| 14.320935 | -91.91243 | *I. coccifer* | *I. coccifer* |
| 14.320935 | -91.91243 | *I. coccifer* | *I. coccifer* |
| 14.320935 | -91.91243 | *I. coccifer* | *I. coccifer* |
| 14.320935 | -91.91243 | *I. coccifer* | *I. coccifer* |
| 14.320935 | -91.91243 | *I. coccifer* | *I. coccifer* |
| 14.325942 | -91.913725 | *I. coccifer* | *I. coccifer* |
| 14.325942 | -91.913725 | *I. coccifer* | *I. coccifer* |
| 14.325942 | -91.913725 | *I. coccifer* | *I. coccifer* |
| 14.325942 | -91.913725 | *I. coccifer* | *I. coccifer* |
| 14.325942 | -91.913725 | *I. coccifer* | *I. coccifer* |
| 14.223417 | -90.147145 | *I. coccifer* | *I. coccifer* |
| 11.483676 | -85.559265 | *I. coccifer* | *I. coccifer* |
| 11.906393 | -85.915398 | *I. coccifer* | *I. coccifer* |
| 12.163883 | -86.142838 | *I. coccifer* | *I. coccifer* |
| 12.163883 | -86.142838 | *I. coccifer* | *I. coccifer* |
| 12.163883 | -86.142838 | *I. coccifer* | *I. coccifer* |
| 12.163883 | -86.142838 | *I. coccifer* | *I. coccifer* |
| 12.163883 | -86.142838 | *I. coccifer* | *I. coccifer* |
| 12.206155 | -86.103053 | *I. coccifer* | *I. coccifer* |
| 11.541592 | -85.945336 | *I. coccifer* | *I. coccifer* |
| 14.547125 | -83.99829 | *I. coccifer* | *I. coccifer* |
| 14.609592 | -83.944591 | *I. coccifer* | *I. coccifer* |
| 13.267276 | -87.644555 | *I. coccifer* | *I. coccifer* |
| 14.31196 | -88.099483 | *I. porteri* | *I. coccifer* |
| 13.267276 | -87.644555 | *I. porteri* | *I. coccifer* |
| 14.6032 | -87.999802 | *I. porteri* | *I. porteri* |
| 14.6297998 | -87.912498 | *I. porteri* | *I. porteri* |
| 14.6297998 | -87.912498 | *I. porteri* | *I. porteri* |
| 14.63933 | -87.91806 | *I. porteri* | *I. porteri* |
| 14.6501999 | -87.838402 | *I. porteri* | *I. porteri* |
| 14.5 | -87.5 | *I. porteri* | *I. porteri* |
| 13.8696003 | -87.248199 | *I. porteri* | *I. porteri* |
| 14.0167 | -87.0833 | *I. porteri* | *I. porteri* |
| 14.09343 | -87.09127 | *I. porteri* | *I. porteri* |
| 14.09343 | -87.09127 | *I. porteri* | *I. porteri* |
| 14.17175 | -87.30732 | *I. porteri* | *I. porteri* |
| 14.17175 | -87.30732 | *I. porteri* | *I. porteri* |
| 14.17175 | -87.30732 | *I. porteri* | *I. porteri* |
| 14.17175 | -87.30732 | *I. porteri* | *I. porteri* |
| 14.17175 | -87.30732 | *I. porteri* | *I. porteri* |
| 14.17175 | -87.30732 | *I. porteri* | *I. porteri* |
| 14.26667 | -87.44634 | *I. porteri* | *I. porteri* |
| 14.18558 | -87.08933 | *I. porteri* | *I. porteri* |
| 14.18558 | -87.08933 | *I. porteri* | *I. porteri* |
| 14.18558 | -87.08933 | *I. porteri* | *I. porteri* |
| 14.18558 | -87.08933 | *I. porteri* | *I. porteri* |
| 14.18558 | -87.08933 | *I. porteri* | *I. porteri* |
| 14.18558 | -87.08933 | *I. porteri* | *I. porteri* |
| 14.0167 | -87.0833 | *I. porteri* | *I. porteri* |
| 14.0167 | -87.0833 | *I. porteri* | *I. porteri* |
| 14.0167 | -87.0833 | *I. porteri* | *I. porteri* |
| 14.0167 | -87.0833 | *I. porteri* | *I. porteri* |
| 14.0167 | -87.0833 | *I. porteri* | *I. porteri* |
| 14.0167 | -87.0833 | *I. porteri* | *I. porteri* |
| 14.0167 | -87.0833 | *I. porteri* | *I. porteri* |
| 14.0167 | -87.0833 | *I. porteri* | *I. porteri* |
| 14.0167 | -87.0833 | *I. porteri* | *I. porteri* |
| 14.0167 | -87.0833 | *I. porteri* | *I. porteri* |
| 14.0167 | -87.0833 | *I. porteri* | *I. porteri* |
| 14.0167 | -87.0833 | *I. porteri* | *I. porteri* |
| 14.0167 | -87.0833 | *I. porteri* | *I. porteri* |
| 14.02477 | -87.08148 | *I. porteri* | *I. porteri* |
| 14.02477 | -87.08148 | *I. porteri* | *I. porteri* |
| 14.18558 | -87.08933 | *I. porteri* | *I. porteri* |
| 14.0833 | -88.1167 | *I. porteri* | *I. porteri* |
| 14.25 | -87.85 | *I. porteri* | *I. porteri* |
| 14.664016 | -87.947416 | *I. porteri* | *I. porteri* |
| 14.664016 | -87.947416 | *I. porteri* | *I. porteri* |
| 14.512316 | -87.879798 | *I. porteri* | *I. porteri* |
| 14.505739 | -87.892025 | *I. porteri* | *I. porteri* |
| 14.091147 | -87.452583 | *I. porteri* | *I. porteri* |
| 14.029782 | -87.074117 | *I. porteri* | *I. porteri* |
| 13.993687 | -86.920867 | *I. porteri* | *I. porteri* |
| 13.993687 | -86.920867 | *I. porteri* | *I. porteri* |
| 14.031017 | -87.074832 | *I. porteri* | *I. porteri* |
| 14.031433 | -87.085206 | *I. porteri* | *I. porteri* |
| 14.034735 | -87.065131 | *I. porteri* | *I. porteri* |
| 14.209852 | -87.09001 | *I. porteri* | *I. porteri* |
| 14.209852 | -87.09001 | *I. porteri* | *I. porteri* |
| 14.209852 | -87.09001 | *I. porteri* | *I. porteri* |
| 14.131103 | -88.051536 | *I. porteri* | *I. porteri* |
| 14.131103 | -88.051536 | *I. porteri* | *I. porteri* |
| 14.131103 | -88.051536 | *I. porteri* | *I. porteri* |
| 14.131103 | -88.051536 | *I. porteri* | *I. porteri* |
| 14.127017 | -88.04351 | *I. porteri* | *I. porteri* |
| 14.128138 | -88.013455 | *I. porteri* | *I. porteri* |
| 14.128138 | -88.013455 | *I. porteri* | *I. porteri* |
| 14.128138 | -88.013455 | *I. porteri* | *I. porteri* |
| 14.128138 | -88.013455 | *I. porteri* | *I. porteri* |
| 14.128138 | -88.013455 | *I. porteri* | *I. porteri* |
| 14.128138 | -88.013455 | *I. porteri* | *I. porteri* |
| 14.127017 | -88.04351 | *I. porteri* | *I. porteri* |
| 14.127017 | -88.04351 | *I. porteri* | *I. porteri* |
| 14.127017 | -88.04351 | *I. porteri* | *I. porteri* |
| 14.127017 | -88.04351 | *I. porteri* | *I. porteri* |
| 14.127017 | -88.04351 | *I. porteri* | *I. porteri* |
| 14.138363 | -88.052246 | *I. porteri* | *I. porteri* |
| 14.138363 | -88.052246 | *I. porteri* | *I. porteri* |
| 14.138363 | -88.052246 | *I. porteri* | *I. porteri* |
| 14.138363 | -88.052246 | *I. porteri* | *I. porteri* |
| 14.138363 | -88.052246 | *I. porteri* | *I. porteri* |
| 14.138363 | -88.052246 | *I. porteri* | *I. porteri* |
| 14.138363 | -88.052246 | *I. porteri* | *I. porteri* |
| 14.138363 | -88.052246 | *I. porteri* | *I. porteri* |
| 14.7991 | -87.84105 | *I. porteri* | *I. porteri* |
| 14.7742666 | -87.84676 | *I. porteri* | *I. porteri* |
| 14.780083 | -87.8462 | *I. porteri* | *I. porteri* |
| 14.771187 | -87.88838 | *I. porteri* | *I. porteri* |
| 14.034904 | -87.076059 | *I. porteri* | *I. porteri* |
| 14.034904 | -87.076059 | *I. porteri* | *I. porteri* |
| 14.034904 | -87.076059 | *I. porteri* | *I. porteri* |
| 14.034904 | -87.076059 | *I. porteri* | *I. porteri* |
| 14.034904 | -87.076059 | *I. porteri* | *I. porteri* |
| 14.034904 | -87.076059 | *I. porteri* | *I. porteri* |
| 14.034904 | -87.076059 | *I. porteri* | *I. porteri* |
| 14.11922 | -87.82989 | *I. porteri* | *I. porteri* |
| 14.14654 | -87.84424 | *I. porteri* | *I. porteri* |
| 14.30463 | -88.18425 | *I. ibarrai* | *I. porteri* |
| 14.30463 | -88.18425 | *I. ibarrai* | *I. porteri* |
| 14.44488 | -88.30748 | *I. ibarrai* | *I. porteri* |
| 14.44488 | -88.30748 | *I. ibarrai* | *I. porteri* |
| 14.44488 | -88.30748 | *I. ibarrai* | *I. porteri* |
| 14.44488 | -88.30748 | *I. ibarrai* | *I. porteri* |
| 14.44488 | -88.30748 | *I. ibarrai* | *I. porteri* |
| 14.43145 | -88.32076 | *I. ibarrai* | *I. porteri* |
| 14.3 | -88.183 | *I. ibarrai* | *I. porteri* |
| 14.3 | -88.183 | *I. ibarrai* | *I. porteri* |
| 14.3 | -88.183 | *I. ibarrai* | *I. porteri* |
| 14.3 | -88.183 | *I. ibarrai* | *I. porteri* |
| 14.3 | -88.183 | *I. ibarrai* | *I. porteri* |
| 14.3 | -88.183 | *I. ibarrai* | *I. porteri* |
| 14.46425 | -88.35177 | *I. ibarrai* | *I. porteri* |
| 14.46425 | -88.35177 | *I. ibarrai* | *I. porteri* |
| 14.46425 | -88.35177 | *I. ibarrai* | *I. porteri* |
| 14.46425 | -88.35177 | *I. ibarrai* | *I. porteri* |
| 14.46425 | -88.35177 | *I. ibarrai* | *I. porteri* |
| 14.41568 | -88.30196 | *I. ibarrai* | *I. porteri* |
| 14.41568 | -88.30196 | *I. ibarrai* | *I. porteri* |
| 14.41568 | -88.30196 | *I. ibarrai* | *I. porteri* |
| 14.2667 | -88.2167 | *I. ibarrai* | *I. porteri* |
| 14.2667 | -88.2167 | *I. ibarrai* | *I. porteri* |
| 14.35 | -88.3667 | *I. ibarrai* | *I. porteri* |
| 14.35 | -88.3667 | *I. ibarrai* | *I. porteri* |
| 14.5667 | -88.6333 | *I. ibarrai* | *I. porteri* |
| 14.43333 | -88.99677 | *I. ibarrai* | *I. porteri* |
| 14.43333 | -88.99677 | *I. ibarrai* | *I. porteri* |
| 14.43333 | -88.99677 | *I. ibarrai* | *I. porteri* |
| 14.43333 | -89.08632 | *I. ibarrai* | *I. porteri* |
| 14.43333 | -89.08632 | *I. ibarrai* | *I. porteri* |
| 14.43333 | -89.08632 | *I. ibarrai* | *I. porteri* |
| 14.43333 | -89.08632 | *I. ibarrai* | *I. porteri* |
| 14.43333 | -89.08632 | *I. ibarrai* | *I. porteri* |
| 14.43333 | -89.08632 | *I. ibarrai* | *I. porteri* |
| 14.4833 | -88.8 | *I. ibarrai* | *I. porteri* |
| 14.5 | -89.3 | *I. ibarrai* | *I. porteri* |
| 14.5 | -89.3 | *I. ibarrai* | *I. porteri* |
| 14.5 | -89.3 | *I. ibarrai* | *I. porteri* |
| 14.5 | -89.3 | *I. ibarrai* | *I. porteri* |
| 14.91333 | -88.7805 | *I. ibarrai* | *I. porteri* |
| 14.91333 | -88.7805 | *I. ibarrai* | *I. porteri* |
| 14.349149 | -88.305531 | *I. ibarrai* | *I. porteri* |
| 14.233398 | -88.466131 | *I. ibarrai* | *I. porteri* |
| 14.233398 | -88.466131 | *I. ibarrai* | *I. porteri* |
| 14.514713 | -88.785696 | *I. ibarrai* | *I. porteri* |
| 14.514713 | -88.785696 | *I. ibarrai* | *I. porteri* |
| 14.421867 | -89.122193 | *I. ibarrai* | *I. porteri* |
| 14.421867 | -89.122193 | *I. ibarrai* | *I. porteri* |
| 14.421867 | -89.122193 | *I. ibarrai* | *I. porteri* |
| 14.421867 | -89.122193 | *I. ibarrai* | *I. porteri* |
| 14.421867 | -89.122193 | *I. ibarrai* | *I. porteri* |
| 14.7991 | -87.84105 | *I. ibarrai* | *I. porteri* |
| 14.7991 | -87.84105 | *I. ibarrai* | *I. porteri* |
| 14.7991 | -87.84105 | *I. ibarrai* | *I. porteri* |
| 14.7284 | -87.9009 | *I. ibarrai* | *I. porteri* |
| 14.11992 | -87.82989 | *I. ibarrai* | *I. porteri* |
| 14.11992 | -87.82989 | *I. ibarrai* | *I. porteri* |
| 14.11992 | -87.82989 | *I. ibarrai* | *I. porteri* |
| 14.11992 | -87.82989 | *I. ibarrai* | *I. porteri* |
| 14.11992 | -87.82989 | *I. ibarrai* | *I. porteri* |
| 14.11992 | -87.82989 | *I. ibarrai* | *I. porteri* |
| 14.11992 | -87.82989 | *I. ibarrai* | *I. porteri* |
| 14.11992 | -87.82989 | *I. ibarrai* | *I. porteri* |
| 14.11992 | -87.82989 | *I. ibarrai* | *I. porteri* |
| 14.11992 | -87.82989 | *I. ibarrai* | *I. porteri* |
| 14.11992 | -87.82989 | *I. ibarrai* | *I. porteri* |
| 14.14654 | -87.84424 | *I. ibarrai* | *I. porteri* |
| 14.14654 | -87.84424 | *I. ibarrai* | *I. porteri* |
| 14.31311 | -88.103352 | *I. ibarrai* | *I. porteri* |
| 14.441386 | -88.145047 | *I. ibarrai* | *I. porteri* |
| 14.441386 | -88.145047 | *I. ibarrai* | *I. porteri* |
| 15.07148 | -90.1 | *I. ibarrai* | *I. ibarrai* |
| 15.089 | -90.03124 | *I. ibarrai* | *I. ibarrai* |
| 15.089474 | -90.119552 | *I. ibarrai* | *I. ibarrai* |
| 15.089474 | -90.119552 | *I. ibarrai* | *I. ibarrai* |
| 15.089474 | -90.119552 | *I. ibarrai* | *I. ibarrai* |
| 15.18819 | -90.198416 | *I. ibarrai* | *I. ibarrai* |
| 15.23774 | -90.272305 | *I. ibarrai* | *I. ibarrai* |
| 15.23774 | -90.272305 | *I. ibarrai* | *I. ibarrai* |
| 15.23774 | -90.272305 | *I. ibarrai* | *I. ibarrai* |
| 15.23774 | -90.272305 | *I. ibarrai* | *I. ibarrai* |
| 15.23774 | -90.272305 | *I. ibarrai* | *I. ibarrai* |
| 15.23774 | -90.272305 | *I. ibarrai* | *I. ibarrai* |
| 15.256577 | -90.259178 | *I. ibarrai* | *I. ibarrai* |
| 15.256577 | -90.259178 | *I. ibarrai* | *I. ibarrai* |
| 14.99896 | -90.807487 | *I. ibarrai* | *I. ibarrai* |
| 14.475295 | -90.663773 | *I. ibarrai* | *I. ibarrai* |
| 14.527159 | -90.60902 | *I. ibarrai* | *I. ibarrai* |
| 14.527159 | -90.60902 | *I. ibarrai* | *I. ibarrai* |
| 14.527159 | -90.60902 | *I. ibarrai* | *I. ibarrai* |
| 14.527159 | -90.60902 | *I. ibarrai* | *I. ibarrai* |
| 14.527159 | -90.60902 | *I. ibarrai* | *I. ibarrai* |
| 14.527159 | -90.60902 | *I. ibarrai* | *I. ibarrai* |
| 14.450443 | -90.584001 | *I. ibarrai* | *I. ibarrai* |
| 14.450443 | -90.584001 | *I. ibarrai* | *I. ibarrai* |
| 14.450443 | -90.584001 | *I. ibarrai* | *I. ibarrai* |
| 14.450443 | -90.584001 | *I. ibarrai* | *I. ibarrai* |
| 15.352505 | -91.313679 | *I. ibarrai* | *I. ibarrai* |
| 15.129168 | -91.534854 | *I. ibarrai* | *I. ibarrai* |
| 15.316266 | -91.542503 | *I. ibarrai* | *I. ibarrai* |
| 15.316266 | -91.542503 | *I. ibarrai* | *I. ibarrai* |
| 15.316266 | -91.542503 | *I. ibarrai* | *I. ibarrai* |
| 15.316266 | -91.542503 | I. ibarrai | I. ibarrai |
| 15.316266 | -91.542503 | I. ibarrai | I. ibarrai |
| 15.316266 | -91.542503 | I. ibarrai | I. ibarrai |
| 15.514315 | -91.870315 | I. ibarrai | I. ibarrai |
| 15.514315 | -91.870315 | I. ibarrai | I. ibarrai |
| 15.514315 | -91.870315 | I. ibarrai | I. ibarrai |
| 15.514315 | -91.870315 | I. ibarrai | I. ibarrai |
| 15.514315 | -91.870315 | I. ibarrai | I. ibarrai |
| 14.69325 | -90.003305 | I. ibarrai | I. ibarrai |
| 14.61389 | -89.934537 | I. ibarrai | I. ibarrai |
| 14.61389 | -89.934537 | I. ibarrai | I. ibarrai |
| 14.61389 | -89.934537 | I. ibarrai | I. ibarrai |
| 14.61389 | -89.934537 | I. ibarrai | I. ibarrai |
| 14.531054 | -90.828745 | I. ibarrai | I. ibarrai |
| 14.516881 | -90.826863 | I. ibarrai | I. ibarrai |
| 14.516881 | -90.826863 | I. ibarrai | I. ibarrai |
| 14.516881 | -90.826863 | I. ibarrai | I. ibarrai |

**Figure S1.** Population assignment for 64 individuals based on *(a)* hierarchical Bayesian population clustering using structure and *(b)* maximum-likelihood population clustering using admixture. The spatial distribution of populations is presented with the same color scheme as figure 1b in the main manuscript.

**Figure S2.** Posterior distribution of genealogical divergence indexes (*gdi*) estimated from the BPP parameters.

**Figure S3.** Heatmaps of (*a*) bioclim variable and (*b*) ENVIREM variable correlation.

**Figure S4.** Continuous outputs from the species distribution models of *I. coccifer*, *I. ibarrai*, and *I. porteri* based on their respective genetic datasets. Warmer colors indicate areas of high probability of occurrence, whereas cooler colors indicate a lower probability of occurrence.

**Figure S5.** Map of the proposed distributions for the focal taxa (*I. coccifer* = red; *I. ibarrai* = yellow; *I. porteri* = blue) with sampling localities that correspond to our genetic, morphological, and macroecological modeling analyses. Dashed lines represent the borders of the Chortís Block, which serve as biogeographic barriers to highland taxa *I. ibarrai* and *I. porteri*.
